# Single-Electron Transistor Based on Quantum Dots in Twisted Graphene/Hexagonal Boron Nitride Bilayer Heterostructure

**DOI:** 10.3390/molecules31050828

**Published:** 2026-03-01

**Authors:** Xinyu Wang, Liang Deng, Fuhao Wang, Shengqiang Ding, Fuan Wang, Jiarui Chen, Haolin Lu, Guankui Long, Zhongkai Huang

**Affiliations:** 1 Key Laboratory of Extraordinary Bond Engineering and Advanced Materials Technology of Chongqing, Yangtze Normal University, Chongqing 408100, China; 2 School of Materials Science and Engineering, National Institute for Advanced Materials, Renewable Energy Conversion and Storage Center (RECAST), Nankai University, Tianjin 300350, China

**Keywords:** graphene/hexagonal boron nitride heterojunction, single-electron transistor, stacking configuration regulation, twist angle effect, quantum dot size

## Abstract

Twisted graphene/hexagonal boron nitride (TG/hBN) bilayers, with their tunable moiré potential and atomically clean interfaces, offer an ideal platform for high-performance single-electron transistors (SET). Combining quantum transport simulations with first-principles calculations, we systematically investigate how stackings (AA, AB, BA), twist angles, quantum dot sizes, and gate-island coupling jointly modulate SET performance. Our central finding reveals a clear hierarchy: quantum dot size and stacking configuration dominate charge stability and transport, while twist angle introduces precise control of charge state. All stackings exhibit sharp, symmetric Coulomb blockade peaks, confirming stable single-electron tunneling, and gate coupling remains highly linear across parameters. Strikingly, only AA-stacked devices show a systematic twist-angle-dependent shift in conductance peaks, a direct signature of its perfect atomic registry and extreme angular sensitivity. This work establishes an idealized “size-, stacking-, and twist-angle modulation” design principle and theoretical roadmap based on TG/hBN, providing fundamental insights for future experimental exploration of tunable, low-noise quantum-electronic devices from twisted 2D heterostructures.

## 1. Introduction

Van der Waals heterostructures composed of graphene and hexagonal boron nitride (hBN) have become one of the multifunctional bilayer platforms for studying the electronic properties of two-dimensional materials [1,2,3]. The hBN has a wide band gap, atomically smooth surface, and excellent dielectric shielding properties, which can effectively reduce substrate-induced disorder and charge inhomogeneity in distribution, providing a near-ideal support and encapsulation environment for graphene [1,4,5,6]. It also significantly suppresses the shot noise originating from substrate impurities [7], and this feature serves as a core advantage for fabricating low-noise quantum electronic devices. Based on this, graphene/hBN bilayer heterostructures can not only maintain the intrinsic high carrier mobility of graphene, but also significantly suppress surface roughness scattering and Coulomb impurity scattering, making them play a key role in the study of quantum Hall effect, quantum spin-correlated transport, and low-dimensional electronic correlation systems [2,8,9,10]. In recent years, with the continuous advancement of two-dimensional material device fabrication technology, including the maturity of methods such as dry transfer, bubble-free encapsulation and high-precision angle alignment, high-quality graphene/hBN heterojunctions have been able to be stably and reproducibly fabricated, which has further promoted their application in nanoelectronics and quantum devices [1,11,12,13,14,15].

When a relative twist angle is introduced between monolayer graphene and hBN, a twisted graphene/hBN (TG/hBN) heterostructure is formed, and a moiré superlattice is generated under the action of interlayer interaction, which significantly modulates the band structure of graphene [16,17,18,19]. Unlike the flat band physics formed by the electronic coupling of two layers of bilayer twisted graphene, the moiré band in the TG/hBN system mainly originates from the periodic potential field modulation caused by lattice constant mismatch and twist angle. Its typical features include the appearance of secondary Dirac points, band gap opening, and Fermi velocity renormalization [2,18,20,21,22,23,24,25]. In addition, the presence of hBN will destroy the sublattice symmetry of graphene, introduce an effective mass term and interlayer periodic potential field, and cause the moiré band to be reconfigured, exhibiting enhanced electronic localization characteristics and tunable band structure under specific twist angle conditions [26,27,28].

Quantum dot (QD) systems based on the TG/hBN moiré potential have attracted widespread attention due to their controllable energy level structure, highly localized electronic states, and low defect characteristics brought about by the van der Waals interface [11,19,29]. Existing theoretical and experimental studies have shown that the energy level distribution and electronic state properties of TG/hBN QDs are jointly regulated by multiple factors such as the moiré potential depth, the applied electric field, and the displacement field, and can exhibit tunable band gaps, charge polarization, and varying degrees of electronic localization behavior [17,20,30]. Therefore, TG/hBN QDs not only provide an ideal model system for studying the moiré confinement effect, but also lay an important physical foundation for constructing novel coherent quantum devices and two-dimensional quantum electronics platforms.

Besides the torsion angle and external electric field, the electronic properties of graphene/hBN bilayer structures are also significantly modulated by the local stacking configuration [31,32,33,34]. Since the lattice constants of graphene and hBN differ by only about 1.7%, a small relative displacement can lead to the periodic appearance of different stacking regions such as AA, AB, and BA, forming unique local moiré potential depressions and barrier structures [6,35]. These stacking configurations correspond to different interlayer coupling strengths, different degrees of symmetry breaking, and different local potential energy distributions, causing charge carriers to exhibit completely different electronic behaviors in different regions. Systematic studies have shown that different stackings lead to significant changes in charge distribution, band arrangement, and local density of states, and can even induce localized states, strongly correlated states, or valley-selective properties [23].

Single-electron transistors (SET) provide a powerful research framework for probing discrete charge states in nanoscale systems [36]. The working mechanism of SET is based on the weak tunneling coupling between Coulomb islands and source/drain electrodes, as well as the electrostatic capacitive coupling with the gate. Their transport behavior is controlled by the Coulomb blocking effect; when the charging energy of electrons exceeds the thermal fluctuation energy level, the current exhibits periodic Coulomb oscillations [37,38,39]. Therefore, the performance of SET mainly depends on the size of the Coulomb islands, the band structure, the dielectric shielding environment, and the level of interface impurities. The ultrathin thickness, high capacitive coupling efficiency, and electronic properties that can be flexibly controlled by external fields of two-dimensional materials make them ideal candidate systems for constructing SET Coulomb islands. In particular, TG/hBN bilayers, due to their controllable twist angle, rich moiré band structure, tunable interlayer coupling, and stable dielectric environment, offer a unique advantage for constructing novel, tunable, and low-noise SET devices.

This study investigates SETs based on TG/hBN QDs. By systematically analyzing the effects of twist angle, stacking configuration, and size on the TG/hBN QDs, we aim to uncover the underlying physics governing their energy level structures, localization mechanisms, and charging behaviors. This further elucidates their role as Coulomb islands within the SET architecture. The research not only provides a theoretical foundation for developing novel low-power quantum devices based on twisted two-dimensional materials but also offers key insights into tuning pathways for electron correlations and confinement effects in twisted systems. The discussion is given in Section 2: moiré patterns of TG/hBN are studied in Section 2.1, Section 2.2 explores the effect of stacking orders on the performance of SET, Section 2.3 displays the effect of twist angles, Section 2.4 explains the effect of sizes on the working condition of SET. The computational methods are described in Section 3, and Conclusions are drawn in Section 4.

## 2. Results and Discussions

### 2.1. Selection of Stackings and Twisting Angles

Due to the large rotational degrees of freedom of graphene/hBN, various long-period moiré superlattices can be formed. We selected seven structures of moiré superlattices with twist angles between 0° and 30° as the baseline configuration. For each baseline configuration, we constructed QDs with various radii and stacking orders. For QDs of the same radius and stacking order, the same center position is selected to emphasize the change in twist angles. Before fabricating QDs, we first make clear structures of TG/hBN.

**Figure 1:**
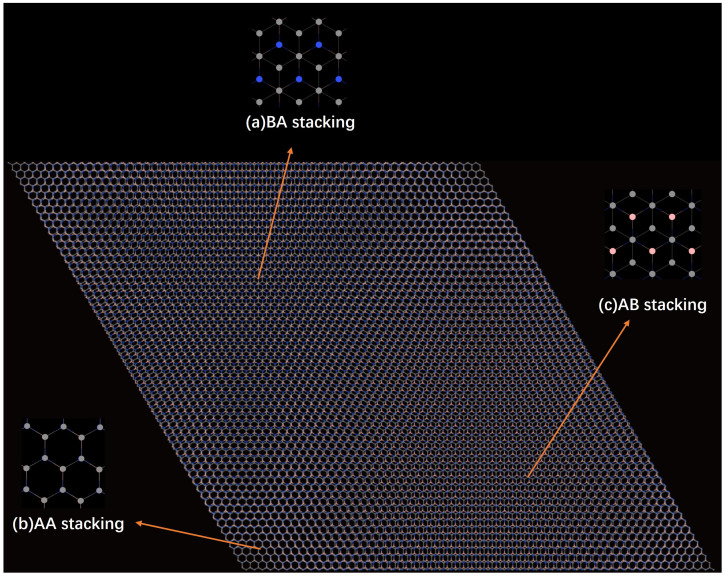
A schematic diagram of the supercell stacking structure of a twisted graphene/hexagonal boron nitride (TG/hBN) bilayer heterojunction. The magnified views of each part correspond to the atomic arrangement of different stacking modes: (**a**) is the local atomic arrangement of BA stacking; (**b**) is the local atomic arrangement of AA stacking; (**c**) is the local atomic arrangement of AB stacking. Gray, blue, and pink balls represent carbon, nitrogen, and boron atoms, respectively.

At the start, a graphene/hBN superlattice with 1.7% lattice mismatch and without twist angles is constructed, as shown in Figure 1. Three typical stacking configurations (AA, AB, BA) can be clearly found in the mismatched moiré pattern.

The interlayer spacing of the graphene/hBN bilayer is set to the equilibrium distance of d = 3.3 Å, while the C-C and B-N bond lengths are set to 1.42 Å and 1.45 Å, respectively. The base vectors in the primitive cells of hBN and graphene are given in the following:

$$\begin{array}{cc} {\vec{a}}_{1} & =\left(\frac{\sqrt{3}}{2},-\frac{1}{2}\right)a_{0},\;{\vec{a}}_{2}=\left(\frac{\sqrt{3}}{2},\frac{1}{2}\right)a_{0},\\ {\vec{b}}_{1} & =\left(\frac{\sqrt{3}}{2},-\frac{1}{2}\right)b_{0},\;{\vec{b}}_{2}=\left(\frac{\sqrt{3}}{2},\frac{1}{2}\right)b_{0}, \end{array}$$

where *a*_0_ and *b*_0_ correspond to the lattice constants of hBN and graphene, respectively. To describe the moiré superstructure of this heterojunction, the basis vectors of the superstructure can be represented by integer combinations of the primitive cell basis vectors of hBN:

$$\begin{array}{cc} {\vec{T}}_{1} & =n{\vec{a}}_{1}+m{\vec{a}}_{2},\;{\vec{T}}_{2}=-m{\vec{a}}_{1}+\left(m+n\right){\vec{a}}_{2}. \end{array}$$

To achieve lattice commensurability between graphene and the hBN superstructure (a prerequisite for the subsequent rotation operation), we simultaneously construct two corresponding vectors in the primitive cell of graphene, which take the form:

$$\begin{array}{cc} {\vec{t}}_{1} & =p{\vec{b}}_{1}+q{\vec{b}}_{2},\;{\vec{t}}_{2}=-q{\vec{b}}_{1}+\left(p+q\right){\vec{b}}_{2}. \end{array}$$

To achieve commensurate matching between the two lattices, we rotate graphene relative to the hBN substrate, such that the superstructure basis vectors ${\vec{T}}_{1}$, ${\vec{T}}_{2}$ coincide with the graphene vectors ${\vec{t}}_{1}$, ${\vec{t}}_{2}$ one-to-one; at this point, the relative rotation angle θ between the two lattices can be calculated via the dot product and modulus of the vectors, with the formula:

$$\begin{array}{cc} \cos\theta & =\frac{{\vec{T}}_{1}\cdot {\vec{t}}_{1}}{|{\vec{T}}_{1}\left|\times \right|{\vec{t}}_{1}|}=\frac{np+mq+(nq+mp)/2}{\sqrt{m^{2}+n^{2}+mn}\times \sqrt{p^{2}+q^{2}+pq}} \end{array}$$

To match the lattices of graphene and hBN within a finite supercell, we finely tune the B-N bond length of hBN (for constructing the lattice mismatch model). Parameter definitions are specified as follows [40]: $L_{\mathrm{BN}}$: The reference equilibrium B-N bond length in isolated monolayer hBN (a fixed physical reference value), taking 1.45 Å; $l_{\mathrm{CC}}$: The fixed C-C bond length of graphene in calculations, taking 1.42 Å (it is correlated with the fine-tuned hBN bond length for simplification in the model); $l_{\mathrm{BN}}$: The fine-tuned B-N bond length of hBN (around $L_{\mathrm{BN}}$) used for supercell construction.

We use the parameter Δ to measure the relative change between the fine-tuned bond length $l_{\mathrm{BN}}$ and the reference bond length $L_{\mathrm{BN}}$, which is defined as:

$$\begin{array}{cc} \Delta & =\frac{|l_{\mathrm{BN}}-L_{\mathrm{BN}}|}{L_{\mathrm{BN}}} \end{array}$$

Combined with the model setting ($l_{\mathrm{CC}}$ is fixed at 1.42 Å) and the modulus relationship between the superstructure vector (with modulus $L_{\mathrm{BN}}\sqrt{m^{2}+n^{2}+mn}$) and graphene vector (with modulus $l_{\mathrm{CC}}\sqrt{p^{2}+q^{2}+pq}$), the above relative variation can be further expressed as:

$$\begin{array}{cc} \Delta & =\frac{\left|l_{\mathrm{CC}}\sqrt{p^{2}+q^{2}+pq}-L_{\mathrm{BN}}\sqrt{m^{2}+n^{2}+mn}\right|}{L_{\mathrm{BN}}\sqrt{m^{2}+n^{2}+mn}} \end{array}$$

Table 1 lists structure formation in TG/hBN bilayers for a series of selected twist angles. As shown in the last row of Table 1, the strain induced by twisting is extremely small, indicating that the mismatch between graphene and hBN remains good, and the pristine effect of twist angles dominates the formation of the studied superlattices. In order to properly explore the influence of TG/hBN on nanodevices, circular QDs based on those moiré structures are adopted as islands of SET in the following sections.

### 2.2. Effect of Stacking Configurations

**Figure 2:**
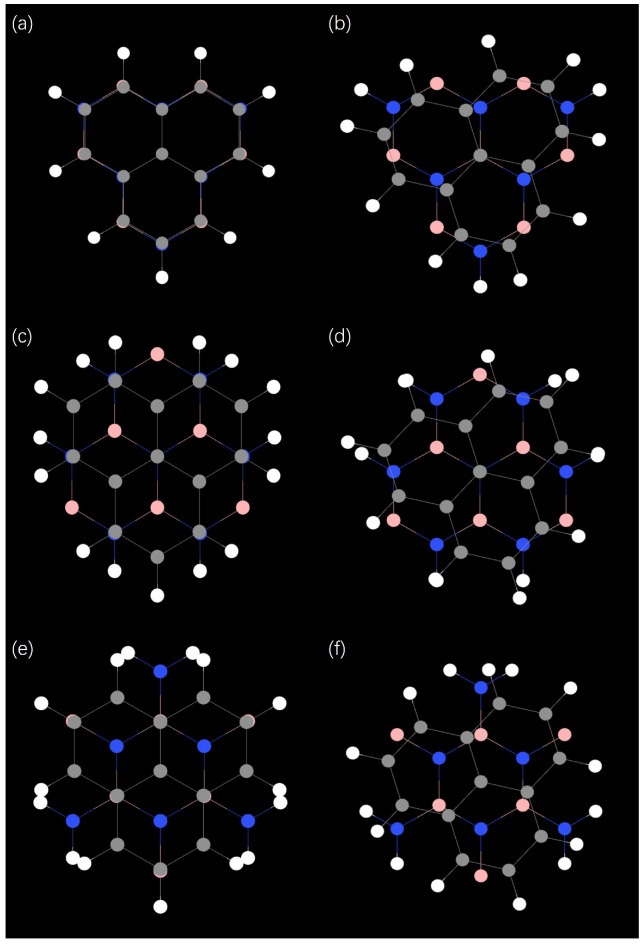
Schematic atomic structures of graphene/hBN bilayer heterostructures with different stacking configurations at 0° (unrotated) and 17.05° twist angles. (**a**–**f**) show the atomic arrangements of graphene/hBN heterostructures for different stacking configurations, where (**a**,**b**) denote AA stacking, (**c**,**d**) denote AB stacking, and (**e**,**f**) denote BA stacking. The gray spheres represent carbon atoms in graphene, the pink and blue spheres represent boron and nitrogen atoms in hBN, respectively, and the white spheres represent hydrogen atoms of the system.

Figure 2a,c,e shows schematic diagrams of three typical stacking (AA, AB, BA) configurations of TG/hBN QDs under the unrotated condition, while Figure 2b,d,f correspond to the case with a twist angle of 17.05°. Figures S1–S6 in the Supporting Information show the QD structures for these three stacking methods at seven specific rotation angles. All models use QD regions with the same center and radius to eliminate the influence of geometric size differences on electron transport properties, thus highlighting the physical effects of the stacking configuration and twist angle itself. It can be seen that there are significant differences between the unrotated and rotated structures in terms of atomic arrangement and interlayer stacking, and these differences are further amplified between different stacking configurations.

**Figure 3:**
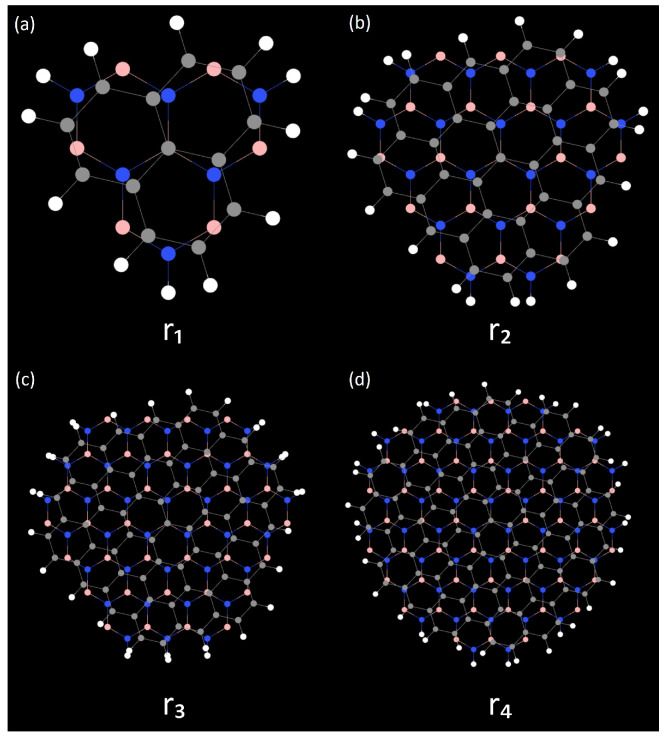
Atomic structures of TG/hBN heterojunction QDs with different sizes. Panels (**a**–**d**) correspond to QDs with increasing radius, denoted as r_1_, r_2_, r_3_, and r_4_, respectively. The radius is defined by the number of concentric hexagonal shells surrounding the central hexagon, ranging from one to four shells.

In the unrotated state, the graphene and hBN interlayer lattice in the AA stack are highly matched, with graphene carbon atoms almost directly overlapping with boron/nitrogen atoms in hBN, exhibiting a highly symmetrical atomic distribution. In contrast, the AB and BA stacks show obvious asymmetric atomic arrangements: in the AB stack, the top-layer graphene carbon atoms are mainly located at the center of the bottom hBN hexagons, while the BA stack is a mirror image of AB. The TG/hBN heterojunction QDs considered in this work adopt a hexagonal geometry with hydrogen-passivated edges. The passivation mechanism is essential for stabilizing the edge nitrogen atoms: in bulk hBN, nitrogen atoms adopt sp^2^ hybridization with three adjacent boron atoms. At the nanosheet edge, a nitrogen atom loses one neighboring boron atom, resulting in one dangling bond with an unpaired electron and one electron-deficient empty orbital. A single hydrogen atom can only saturate the dangling bond, leaving the atom in a metastable state. Two hydrogen atoms enable sp^3^ hybridization, simultaneously saturating the dangling bond and filling the empty orbital, thereby achieving complete electronic passivation of the edge. Their sizes are characterized by the number of concentric hexagonal shells surrounding the central hexagon. Specifically, as shown in Figure 3, four representative QD sizes, denoted as r_1_, r_2_, r_3_, and r_4_, are constructed, corresponding to one, two, three, and four hexagonal shells, respectively. As the number of shells increases, the QDs exhibit a systematic increase in lateral size and carbon atom count, accompanied by a gradual reduction in edge-state dominance. The above stacking configurations and dimensional parameters lay the structural foundation for subsequent electron localization behavior and energy level modulation.

**Figure 4:**
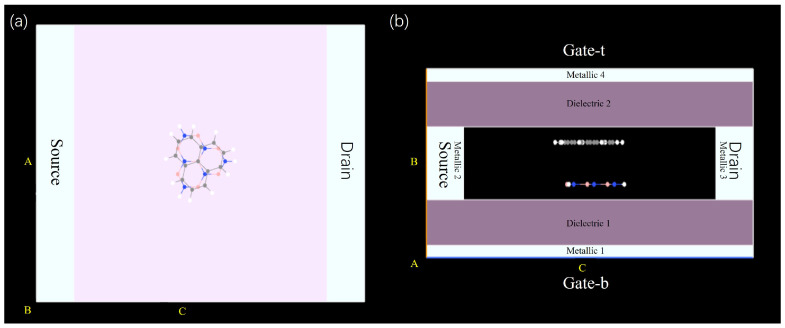
The dual-gate SET structure is shown from (**a**) top-view and (**b**) cross-section view. The configuration is composed of dual gates (Gate-t: top gate, Gate-b: bottom gate), source, drain, and a TG/hBN QD rotated by 17.05°. A, B and C represent first, second and third unit cell vectors of the device. The labels of the dielectric and metallic regions shown in Table 2 correspond to the marked areas in this figure, where the metallic regions correspond to gold gate/source/drain electrodes.

Stacking configuration is a core structural parameter for controlling interlayer coupling, sublattice symmetry, and interface potential distribution in TG/hBN bilayer heterojunctions. For SET based on QDs in such heterojunctions, the stacking order directly modulates quantum confinement effects, charge localization, and electron tunneling behavior. Figure 2 emphasizes stacking/twist-angle effects on structure, while Figure 3 emphasizes size effects on structure. Together, they provide a comprehensive structural basis for understanding the synergistic modulation of SET performance by stacking, twist angle, and size. Figure 4 shows a dual-gate SET structure. This structure introduces another gate, Gate-t, at the top. Gate-t uses the same configuration as Gate-b. This design couples the bottom (top) gate to the TG/hBN QDs, thereby amplifying the impact of the stacking twist angle on the TG/hBN QDs and device performance.

To quantify the spatial extent of these dielectric and metallic regions, their specific parameters are given in Table 2.

This section focuses on three typical stacking configurations (AA, AB, BA). Under the conditions of the same center, radius, and QD size, it comprehensively analyzes the structural characteristics, charge stability, electronic transport properties, and electronic energy level structure by comparing two torsion angle states (0° and 17.05°), and systematically explores their impact on SET performance.

**Figure 5:**
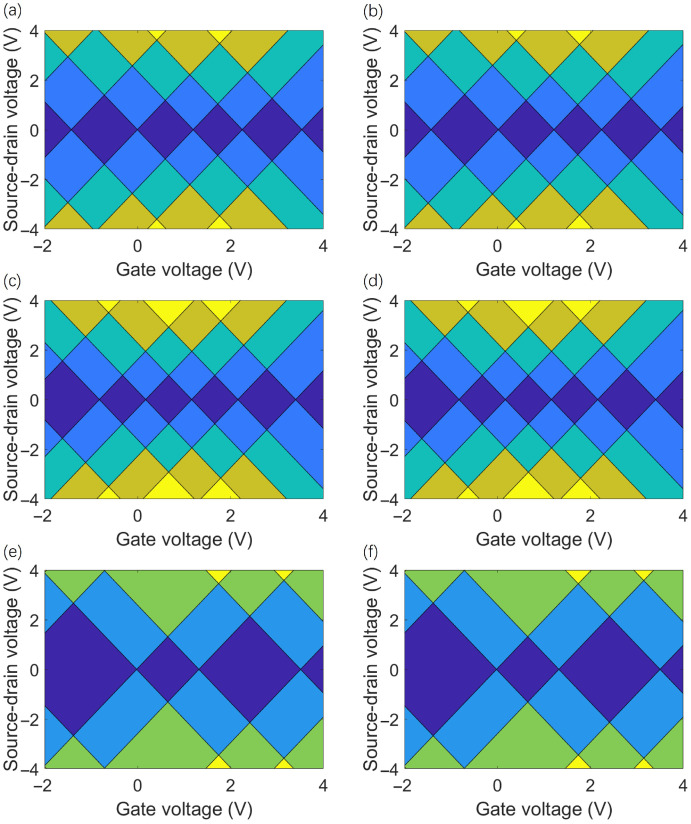
The left (right) column shows the charge stability diagram of the stacked charges AA, AB, BA from top to bottom without rotation (with rotation angle of 17.05°). For (**a**–**d**), the dark blue, blue, teal, olive, and yellow color schemes represent charge state numbers of 0, 1, 2, 3, and 4, respectively; for (**e**,**f**), the dark blue, blue, green, and yellow color schemes mark charge state numbers of 0, 1, 2, and 3, respectively.

Figure 5 shows the charge stability (Coulomb rhombus diagram) of TG/hBN heterojunction QDs, with the horizontal axis representing the gate voltage and the vertical axis representing the source–drain voltage. Figure 5a,c,e correspond to the unrotated (0°) AA, AB, and BA stack configurations, respectively. Figure 5b,d,f shows the results after a 17.05° rotation of the corresponding configurations, covering various charge states. The comparison shows that for the same stack configuration, the outline, symmetry, and charge state dependence of the Coulomb rhombus are not significantly different between the rotated and unrotated states. The core difference stems from the stack configuration itself rather than the rotation angle. The charge stability characteristics of the BA stack configuration are significantly different from those of the AA and AB stack structures. Notably, the 17.05° rotation angle does not significantly change these characteristics; the Coulomb rhombus after rotation for all three stacks is essentially the same as the unrotated state, indicating that the interlayer coupling caused by this rotation angle has little impact on charge stability.

**Figure 6:**
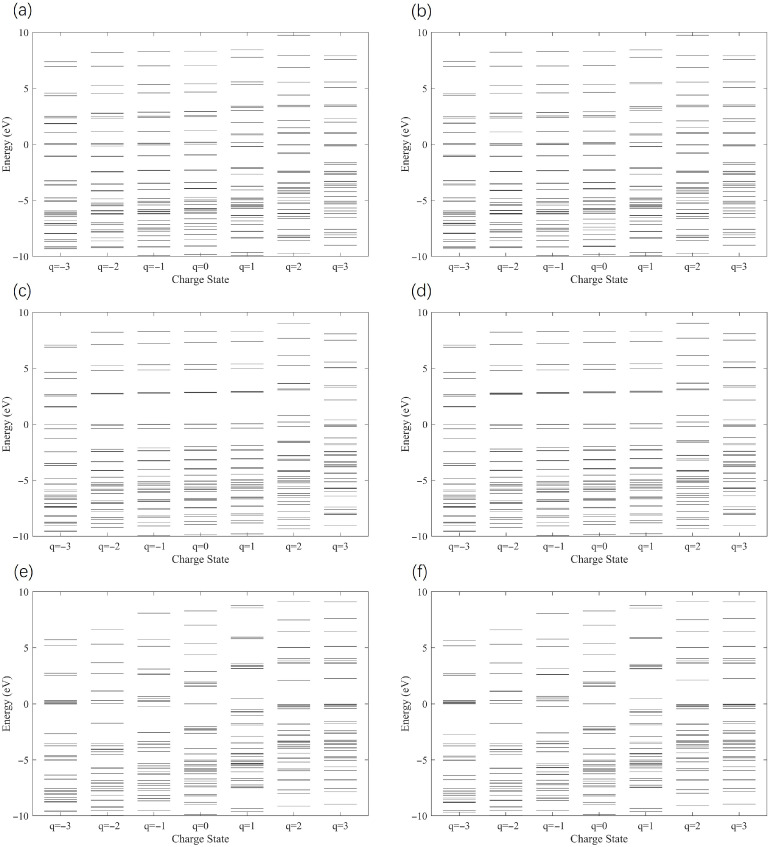
The molecular energy spectra of five charge states (q = −3, −2, −1, 0, 1, 2, and 3) of unrotated and 17.05° rotated TG/hBN bilayer heterojunction QDs in a SET configuration were investigated: spectra of (**a**) AA-unrotated, (**c**) AB-unrotated, (**e**) AB-unrotated; (**b**) AA-17.05°, (**d**) AA-17.05°, (**f**) BA-17.05°.

Figure 6a,c,e shows the molecular spectra of unrotated (0°) AA, AB, and BA stack configurations, respectively. Figure 6b,d,f shows the results after rotating the corresponding stacks by 17.05°. The energy levels of quantum dots exhibit distinct discretization characteristics, which is a core electronic structure signature of Coulomb islands. The regulation of charge states can effectively alter the energy level arrangement of quantum dots, demonstrating that this system meets the key criteria for functioning as a Coulomb island. It can be observed that all six QD structures have relatively clear, separated energy levels. When the TG/hBN heterojunction QDs are negatively charged, the electronic energy levels shift downwards as a whole. This shift creates favorable conditions for the inflow and outflow of net charge in the TG/hBN QDs. Comparing the molecular energy spectra after the 17.05° rotation in Figure 6, it can be seen that the rotation angle did not change the core energy level differences of the three stacks, and the energy level-charge state dependence law of the three is basically consistent with that of the unrotated state. Specifically, the rotation only slightly shrinks the energy level spacing of each stack, without causing energy level reconstruction or the generation of new energy levels. This is consistent with the previous conclusion, indicating that the modulation effect of this rotation angle on interlayer coupling is weak and does not destroy the inherent electronic structure properties of each stack. In summary, molecular energy dispersive spectroscopy further confirms that the inherent symmetry differences in the stacking configuration are the core factors determining the electronic structure, charge stability, and electronic transport properties of TG/hBN QDs. To further analyze the impact of the rotation angle on the performance of SETs, we investigated the gate-island coupling strength for seven rotation angles and three stacking configurations, which will be analyzed in detail in the next subsection.

### 2.3. Effect of Twist Angles

**Figure 7:**
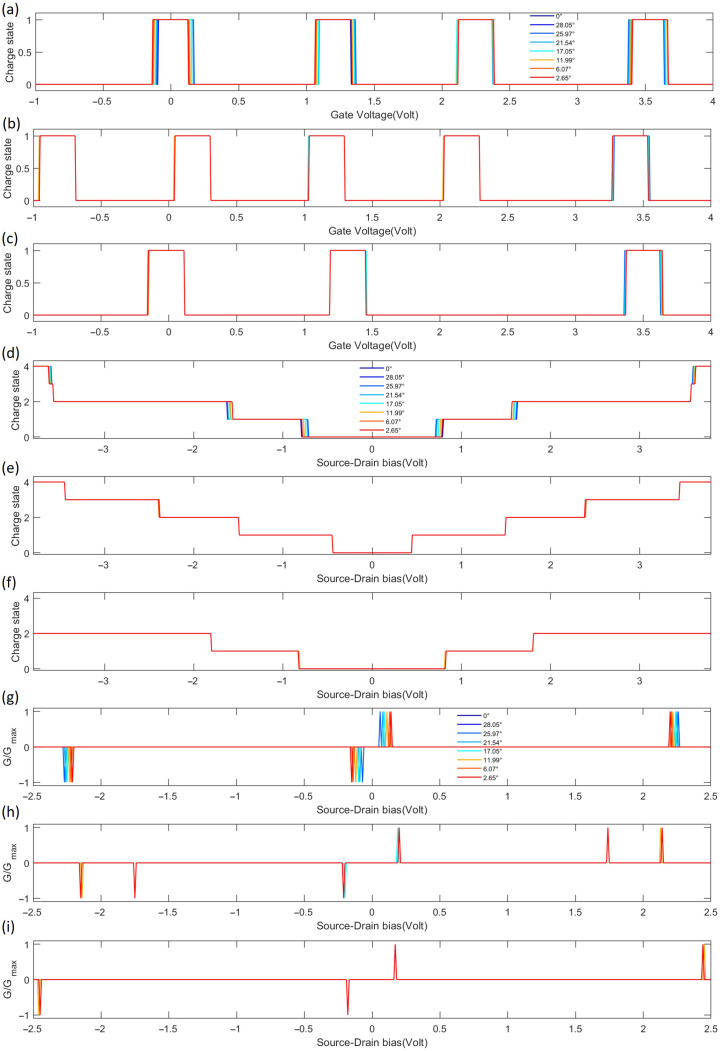
Charge states versus gate voltage, source–drain bias, and conductance versus source–drain bias for SET based on TG/hBN QDs with unrotated and seven rotated angles are presented. Panels (**a**–**c**) correspond to the AA, AB, and BA stacking configurations, respectively, showing the line scans along the gate voltage with the source–drain bias set to 0. Panels (**d**–**f**) correspond to the AA, AB, and BA stacking configurations, respectively, displaying the line scans along the source–drain bias at the maximum of the central rhombus (gate voltage = 3.208 V). Panels (**g**–**i**) correspond to the AA, AB, and BA stacking configurations, respectively, depicting the normalized differential conductance with respect to the source–drain bias (with reference to the scans in (**d**–**f**)).

To characterize the charge stability diagrams, differential conductance line scans were performed across different source–drain and gate voltages, shown in Figure 7a,b,d,e,g,h, and Figure 7c,f,i for the AA, AB, and BA stacking configurations, respectively. To further clarify the impact of stacking configurations on charge transport properties, the key features of these configurations are analyzed below: Each stacking type was measured at a source–drain bias of $V_{\mathrm{sd}}=3.21\;\mathrm{mV}$ across various twist angles. In the gate voltage sweep measurements (Figure 7a–c, $V_{\mathrm{sd}}=0$), the width of charge state plateaus shows a distinct stacking dependence: the AA stacking (Figure 7a) exhibits the narrowest plateaus, corresponding to the smallest charging energy ($E_{C,\mathrm{AA}}$)—attributed to its optimal atomic registry and weakest interlayer coupling; the AB stacking (Figure 7b) has intermediate plateau widths, corresponding to a moderate charging energy ($E_{C,\mathrm{AB}}$); the BA stacking (Figure 7c) features the widest plateaus, corresponding to the largest charging energy ($E_{C,\mathrm{BA}}$)—due to the most significant symmetry breaking and strongest interlayer coupling. Consistent with this trend, source–drain bias sweep measurements (Figure 7d–f), $V_{G}=3.208\;V$) reveal stacking-dependent charge state switching thresholds: the AA stacking (Figure 7d) requires the lowest bias to break Coulomb blockade, the AB stacking (Figure 7e) has an intermediate threshold, and the BA stacking (Figure 7f)—owing to its largest charging energy—demands a higher bias for switching, which is a direct manifestation of its maximum charging energy.

All three stackings exhibit highly symmetric peak shapes, indicating symmetric quantum-dot coupling to the source and drain electrodes with uniform tunnel barriers. Normalized differential conductance spectra (Figure 7g–i) further confirm the stacking effect: the AA stacking (Figure 7g) shows the weakest $dI/dV_{\mathrm{sd}}$ peak intensity (resulting from the widest tunneling barrier associated with the smallest charging energy); the AB stacking (Figure 7h) has intermediate peak intensity; the BA stacking (Figure 7i) exhibits the strongest peak intensity (corresponding to the narrowest tunneling barrier). The uniform peak amplitudes reflect stable sequential electron tunneling as discrete charge states are tuned through the Fermi level by the gate voltage. The sharp differential conductance peaks confirm that the charging energy $E_{C}$ significantly exceeds the thermal fluctuation energy $k_{\;B}T$ which corresponds to 25.9 meV at room temperature (T = 300 K), placing the system in a well-defined Coulomb blockade regime where single-electron tunneling through discrete energy levels is clearly resolved.

Notably, only the AA-stacked devices show a systematic shift in the differential conductance peak positions with varying twist angle, while the AB and BA configurations remain largely unchanged. This distinct behavior carries important physical implications: The AA stacking, with its perfect atomic registry and highest crystal symmetry, renders the electronic structure highly sensitive to the twist angle. Slight variations in the moiré potential significantly alter the electrostatic confinement profile and effective bandwidth of the QDs, thereby shifting its single-electron energy levels—directly manifested as gate-voltage shifts of the conductance peaks. In contrast, AB and BA stackings represent the energetically stable Bernal forms. Their interlayer coupling preserves robust localized electronic characteristics even under small twists, leaving the primary Coulomb blockade spectrum—and hence the peak positions—relatively unaffected by the twist angle [41]. Therefore, the twist angle under the AA stackings exhibits precise tunability over the electronic structure.

The gate-island coupling strength is the core parameter that determines the gate control performance of SET. The variation of its linear coupling coefficient α and quadratic coupling coefficient β comprehensively reflects the essence of the control of device control characteristics by stacking configuration and rotation angle. Table 3 and Table 4 shows the quantitative data of coupling strength for 7 non-zero rotation angles and 3 stacking configurations. The core coupling characteristics of the three configurations can be summarized as follows: The linear coupling coefficient α of the AA stack is stable at 0.9785∼0.9786 with minimal fluctuation, and the quadratic coupling coefficient β shows no significant fluctuation, exhibiting good rotational stability; the BA stack has similar performance to the AA stack, with α stable at ∼0.9783 with minimal fluctuation, and β fixed at −0.000018, also showing excellent rotational stability; in contrast, the linear coupling coefficient α and the quadratic coupling coefficient β of the AB stack exhibit minimal fluctuation, and are relatively more sensitive to rotational disturbances. Meanwhile, the α values of the three stacks are all close to 1 (0.9780∼0.9787), and the β values are all on the order of $10^{-5}\;\mathrm{eV}\cdot V^{-1}$, indicating that the gate’s control over the QDs electrochemical potential is mainly based on efficient linear control with extremely weak nonlinear deviation, providing favorable conditions for precise linear control of SET. Notably, this stable gate-island coupling (reflected in Table 3 and Table 4) is complementary to the twist-angle sensitivity of AA stacking in Figure 7: while AA stacking’s coupling coefficients remain robust (ensuring reliable gate control), its electronic structure (and thus conductance peak positions) is highly sensitive to twist angles. This combination—stable control capability plus tunable electronic states—makes AA-stacked QDs a promising candidate for flexible, high-precision SET devices.

### 2.4. Effect of Quantum Dot Sizes

**Figure 8:**
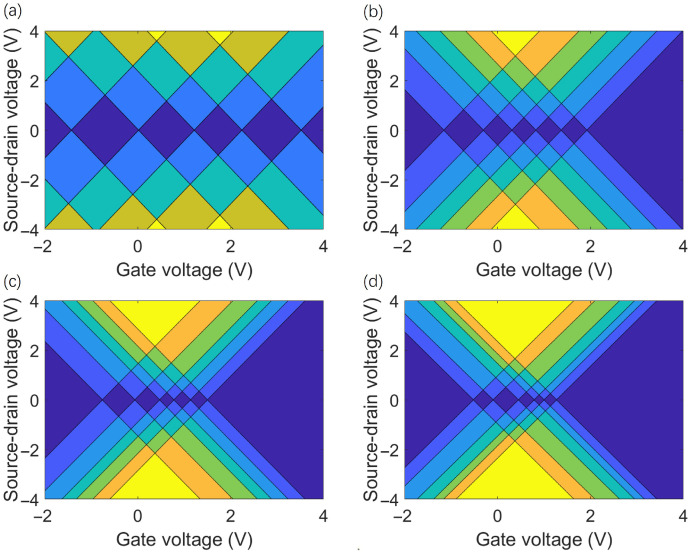
Charge stability diagrams for four AA-stacked structures with different radii (labeled r_1_, r_2_, r_3_, and r_4_ with increasing radii) at a twist angle of 17.05° for various charge states, which denotes the number of net charge on the quantum dots. For (**a**), the dark blue, blue, teal, olive and yellow color schemes represent charge state numbers of 0, 1, 2, 3, and 4, respectively; for (**b**–**d**), the dark blue, blue, royal blue, teal, green, orange and yellow color schemes represent charge state numbers of 0, 1, 2, 3, 4, 5 and 6, respectively.

QD size is a key parameter for tuning quantum confinement effects. This section focuses on the AA-stacked configuration—previously confirmed to exhibit optimal structural symmetry and rotational robustness—and systematically investigates the influence of QD size on the charge stability characteristics of SET. This is achieved by analyzing charge stability diagrams at a 17.05° twist angle for four AA-stacked QDs with increasing radii (labeled r_1_ to r_4_). The results reveal a clear trend: as the QD size increases, the quantum confinement effect weakens. As shown in Figure 8, this leads to a reduction in the overall size of the Coulomb diamonds and a decrease in charge localization. This phenomenon may be attributed to the increased number of atoms in larger dots suppressing discrete energy levels. Furthermore, to explore the impact of dot size on single-electron transport, the linear and quadratic coupling strengths between the gate electrode and the TG/hBN island QDs were systematically compared for different dot sizes (r_2_–r_4_), as detailed in Table 5 and Table 6. The results indicate that the linear gate-island coupling coefficient α decreases significantly with increasing dot radius, while variations caused by different twist angles are relatively minor. This confirms that QDs size is the dominant factor determining gate efficiency. Smaller QDs possess a more concentrated electric field distribution, allowing the gate voltage to modulate the island potential more effectively and thereby enhancing linear coupling.

In contrast, the second-order coupling coefficient β is negative in all cases, and its absolute value increases significantly with the size of the QDs, reflecting a stronger nonlinear potential response in larger QDs. This size-dependent higher-order effect may originate from uneven capacitance distribution or enhanced charge rearrangement. In summary, smaller QDs are advantageous for obtaining strong linear gating and stable Coulomb oscillations, while larger QDs are more prone to introducing nonlinear transport behavior, thus affecting the accuracy and stability of single-electron control.

The design principles and conclusions presented in this work are derived from idealized theoretical models featuring perfect atomic registry, well-defined twist angles, and defect-free structures. In actual fabricated devices, inevitable variations—such as local strain, disorder, fluctuations in stacking registry, and twist-angle inhomogeneity—can alter quantitative features including precise conductance peak positions, coupling strengths, and energy scales. Therefore, the numerical values and specific device characteristics reported here should be interpreted as trends from a controlled theoretical study rather than as direct predictions for any individual experimental device.

Despite the sensitivity of quantitative metrics to structural imperfections, the hierarchy of influencing factors—where quantum dot size and stacking configuration dominate the electrostatic environment and charge stability, while twist angle provides a finer, secondary modulation—is a consequence of basic electrostatic and symmetry considerations. This hierarchy is therefore anticipated to persist across reasonable variations. In particular, the unique sensitivity of the AA-stacked configuration to twist angle, arising from its perfect registry and uniform interlayer coupling, is a distinctive feature that should remain observable as a general trend, even if the exact angular dependence is smoothed by disorder.

## 3. Methods

### 3.1. Theoretical Modeling of Single-Electron Transistors

The theoretical modeling of SET is based on the quantum transport framework in the sequential tunneling regime, focusing on describing charge localization and tunneling behavior of nanoscale island structures under the Coulomb blockade effect. In the weak coupling regime ($\Gamma \tau_{C}\ll 1$, Γ is the tunneling rate, and $\tau_{C}$ is the charge relaxation time), electron transport exhibits a sequential tunneling process, whose dynamics is dominated by the free energy barriers of the system at different charge states *N* [36,37,38,42].

Transitions between charge states must satisfy the thermodynamic critical condition:

(1) $$E^{\mathrm{source}}\left(M\right)+E^{\mathrm{island}}\left(N\right)\geq E^{\mathrm{source}}\left(M-1\right)+E^{\mathrm{island}}\left(N+1\right)$$

This condition describes the energy criterion for the first step of sequential tunneling (electron transfer from the source to the island). A complete current path requires the second step (electron transfer from the island to the drain) to satisfy the complementary thermodynamic condition:

(2) $$E^{\mathrm{drain}}\left(K\right)+E^{\mathrm{island}}\left(N+1\right)\geq E^{\mathrm{drain}}\left(K+1\right)+E^{\mathrm{island}}\left(N\right)$$

Only when both conditions are met can continuous net current flow (electrons do not accumulate on the island). This condition reflects that the transfer of an electron from the source electrode to the molecular island must be accompanied by a reduction in the total energy of the system. The charging energy $\Delta E^{\mathrm{island}}\left(N\right)$ serves as a key parameter determining the transport threshold, which together with the bias voltage *V* and electrode work function *W* constitutes the current conduction condition:

(3) $$e|V|/2\geq \Delta E^{\mathrm{island}}\left(N\right)+W\geq -e\left|V\right|/2$$

Here, *W* (electrode work function) acts as a reference benchmark linking the electrode Fermi level to the vacuum energy level (a universal energy zero). The term $\Delta E^{\mathrm{island}}\left(N\right)+W$ represents the island’s charging energy relative to the electrode Fermi level; the inequality below describes that this relative energy must fall within the bias window of the source/drain for tunneling to occur.

To quantitatively calculate the energy states of molecules in the device environment, first-principles methods combined with the dielectric continuum model are required. By self-consistently solving the Kohn–Sham equation and the Poisson equation:

(4) $$-\nabla \cdot [\varepsilon \left(r\right)\nabla \delta V^{H+ext}\left(r\right)]=\delta n\left(r\right)$$

The interaction between the molecular charge density and the external electrostatic potential can be accurately obtained. The environmental polarization effect is introduced through the dielectric function ε(r) in the Poisson equation, leading to the renormalization of molecular energy levels and a significant reduction in charging energy.

This framework further reveals the combined regulatory effect of the linear coupling term ($\alpha qV_{G}$) and the nonlinear polarization term ($\beta {\left(eV_{G}\right)}^{2}$) on the charge stability diagram by introducing the modulation term of the gate voltage $V_{G}$ on the system energy:

(5) $$E=\alpha qV_{G}+\beta {\left(eV_{G}\right)}^{2}.$$

Within the stated theoretical framework, as long as the system operates in the incoherent regime where transport is dominated by sequential tunneling, the fundamental features of the stability diagram (such as the Coulomb-blockade thresholds) are determined by the island charging energy and the gate-coupling strength. The microscopic mechanisms of electrode coupling and their role in the coherent/incoherent transport crossover serve as an important future research direction.

### 3.2. Computational Methods

In terms of computational methods, for the large-scale TG/hBN moiré quantum dot system with a supercell containing hundreds to tens of thousands of atoms, given the excessively high computational cost of full DFT calculations, this study employs the DFTB [43] method based on DFT-fitted parameters and adopts the “matsci 0–3” Slater–Koster parameter set (this parameter set is derived from systematic DFT calculations of graphene, hBN, and related interface materials). Its reliability in simulating the structure and electronic properties of various materials has been widely validated by a series of studies [44,45,46,47,48,49,50]. This method enables accurate characterization of the electrostatic confinement potential and energy level structure of the system while ensuring computational efficiency. To appropriately describe electrostatic interactions, pseudopotentials and additional charges are introduced in the SET simulations to effectively screen these interactions; Specifically, the dielectric environment model employed in this study is constructed in accordance with the well-established continuum dielectric approach proposed in [36], and is compatible with the electrostatic screening framework adopted in this work. For the Au electrodes defining the metallic regions in Figure 4, a work function of W = 5.28 eV is used for all electrode-related calculations. All calculations are performed using the QuantumATK-2019 software package [51], which allows for convenient integration of the DFTB and Non-Equilibrium Green’s Function (NEGF) methods, providing technical support for simulating the transport behavior of quantum dot-based SETs. Notably, the reliability of QuantumATK in simulating 2D material-based SET has been validated in previous studies [52,53], which further ensures the credibility of the simulation results obtained in this study.

## 4. Conclusions

This study, combining quantum transport theory and first-principles calculations, systematically reveals the synergistic modulation mechanism of stacking configurations (AA, AB, BA), twist angles, and QD size on the performance of SET based on TG/hBN QDs. The results indicate that QD size and stacking configuration are the dominant factors determining the SET charge stability and transport characteristics, while the twist angle introduces weak moiré potential modulation and enables theoretical tuning. A key finding is that only the AA stacking exhibits unique twist-angle sensitivity, with its differential conductance peak positions shifting with angle, which stems from the high symmetry and sensitive response to moiré potential changes due to its perfect atomic registry. Meanwhile, the gate-island coupling demonstrates high linearity and good stability across the entire parameter range, laying the foundation for precise electrical control of the SET. It should also be noticed that the control of defects and edge states is a crucial future research direction. The present study is a theoretical investigation based on idealized structural models with perfect stacking registry and well-defined parameters. The conclusions drawn herein are intended to reveal general trends and design principles (e.g., the hierarchy of influencing factors, the unique sensitivity of AA stacking), rather than to provide quantitative, device-to-device performance predictions. In real experimental systems, variability in stacking, twist angle, and disorder may alter quantitative features, but the dominant physical trends identified are expected to hold due to their origin in fundamental symmetry and electrostatics. This work clarifies the “size, stacking, and twist-angle modulation” design principle, providing key theoretical guidance for constructing tunable, low-noise single-electron devices based on twisted two-dimensional heterostructures.

## Figures and Tables

**Table 1:** Rotation angles (θ) and corresponding structural information: commensurable variables of *m*, *n*, *p* and *q*, number of atoms *N*, and strained lattice mismatch Δ.

θ	$0^{\circ }$	$28.05^{\circ }$	$25.97^{\circ }$	$21.54^{\circ }$	$17.05^{\circ }$	$11.99^{\circ }$	$6.07^{\circ }$	$2.65^{\circ }$
(m,n)	(59,59)	(−17,9)	(−20,−5)	(−36,2)	(−36,−8)	(−23,−29)	(−17,−38)	(−48,−9)
(p,q)	(58,58)	(15,−15)	(7,−26)	(17,−41)	(6,−44)	(−38,−13)	(−34,−23)	(−12,−47)
*N*	13,690	884	2136	5002	6712	8288	9692	11,468
Δ	0.01%	0.04%	0.09%	0.03%	0.02%	0.08%	0.04%	0.03%

**Table 2:** Coordinate ranges and thickness parameters of dielectric and metallic Regions (Region labels correspond to the marked areas in Figure 4).

Region	Coordinate Range (Å)			Thickness (Å)		
	x	y	z	Δx	Δy	Δz
Dielectric 1	[0,27]	[1,4.7]	[0,35]	27	3.7	35
Dielectric 2	[0,27]	[10.6,14.3]	[0,35]	27	3.7	35
Metallic 1	[0,27]	[0,1]	[0,35]	27	1	35
Metallic 2	[0,27]	[4.7,10.6]	[0,4]	27	5.9	4
Metallic 3	[0,27]	[4.7,10.6]	[31,35]	27	5.9	4
Metallic 4	[0,27]	[14.3,15.3]	[0,35]	27	1	35

**Table 3:** The linear coupling strength between the gate and the island of TG/hBN QDs in a double-gated SET with AA, AB, and BA stacking configurations and a radius of r_1_, involving 8 AA stacking rotation angles.

Rotation Angle θ	Linear Gate-Island Coupling Strength (α)		
	AA	AB	BA
$0^{\circ }$	0.978565	0.978093	0.978342
$28.05^{\circ }$	0.978635	0.978293	0.978386
$25.97^{\circ }$	0.978640	0.978286	0.978387
$21.54^{\circ }$	0.978646	0.978286	0.978385
$17.05^{\circ }$	0.978644	0.978246	0.978375
$11.99^{\circ }$	0.978634	0.978213	0.978359
$6.07^{\circ }$	0.978610	0.978158	0.978346
$2.65^{\circ }$	0.978588	0.978122	0.978343

**Table 4:** The quadratic coupling strength between the gate and the island of TG/hBN QDs in a double-gated SET with AA, AB, and BA stacking configurations and a radius of r_1_, involving 8 AA stacking rotation angles.

Rotation Angle θ	Linear Gate-Island Coupling Strength (β(eV^−1^))		
	AA	AB	BA
$0^{\circ }$	−0.000019	−0.000022	−0.000018
$28.05^{\circ }$	−0.000019	−0.000018	−0.000018
$25.97^{\circ }$	−0.000019	−0.000019	−0.000018
$21.54^{\circ }$	−0.000019	−0.000019	−0.000018
$17.05^{\circ }$	−0.000019	−0.000020	−0.000018
$11.99^{\circ }$	−0.000019	−0.000020	−0.000018
$6.07^{\circ }$	−0.000019	−0.000021	−0.000018
$2.65^{\circ }$	−0.000019	−0.000022	−0.000018

**Table 5:** The linear coupling strength between the gate and the island of TG/hBN QDs in a double-gated SET with an AA stacking configuration, which involves 8 AA stacking rotation angles and 3 QDs radii r_2_/r_3_/r_4_.

Rotation Angle θ	Linear Gate-Island Coupling Strength (α)		
	r_2_	r_3_	r_4_
$0^{\circ }$	0.968556	0.948336	0.919285
$28.05^{\circ }$	0.968788	0.948860	0.919341
$25.97^{\circ }$	0.968790	0.948847	0.919509
$21.54^{\circ }$	0.968788	0.948791	0.919677
$17.05^{\circ }$	0.968772	0.948730	0.919728
$11.99^{\circ }$	0.968738	0.948664	0.919697
$6.07^{\circ }$	0.968682	0.948591	0.919615
$2.65^{\circ }$	0.968626	0.948535	0.919508

**Table 6:** The quadratic coupling strength between the gate and the island of TG/hBN QDs in a double-gated SET with an AA stacking configuration, involving 8 AA stacking rotation angles and 3 QDs radii r_2_/r_3_/r_4_.

Rotation Angle θ	Linear Gate-Island Coupling Strength (β(eV^−1^))		
	r_2_	r_3_	r_4_
$0^{\circ }$	−0.000285	−0.002341	−0.011073
$28.05^{\circ }$	−0.000279	−0.002331	−0.010834
$25.97^{\circ }$	−0.000279	−0.002326	−0.010739
$21.54^{\circ }$	−0.000278	−0.002317	−0.010656
$17.05^{\circ }$	−0.000278	−0.002310	−0.010670
$11.99^{\circ }$	−0.000278	−0.002304	−0.010739
$6.07^{\circ }$	−0.000280	−0.002306	−0.010856
$2.65^{\circ }$	−0.000282	−0.002312	−0.010957

## Data Availability

Data are contained within the article. Further inquiries can be directed to the corresponding author.

## Supplementary Materials

The following supporting information can be downloaded at: https://www.mdpi.com/article/10.3390/molecules31050828/s1.
