# Supplementary material for: Single-Electron Transistor Based on Quantum Dots in Twisted Graphene/Hexagonal Boron Nitride Bilayer Heterostructure
Source: Molecules. 2026 Mar 1;31(5):828. doi: 10.3390/molecules31050828 (PMC12986261; doi:10.3390/molecules31050828)
Supplement: Supplementary file 1 [file molecules-31-00828-s001.zip › molecules-4098988-supplementary.pdf]

Supporting Information

Single-Electron Transistor Based on Quantum Dots in Twisted Graphene/Hexagonal Boron Nitride Bilayer Heterostructure

Xinyu Wang [1], Liang Deng [1], Fuhao Wang [1], Shengqiang Ding [1], Fuan Wang [1], Jiarui Chen [1], Haolin Lu[2], Guankui Long [2]*, and Zhongkai Huang [1]*

[1]*Key Laboratory of Extraordinary Bond Engineering and Advanced Materials Technology of Chongqing, Yangtze Normal University, Chongqing 408100, China*

[2]*School of Materials Science and Engineering, National Institute for Advanced Materials, Renewable Energy Conversion and Storage Center (RECAST), Nankai University, Tianjin 300350, China*

(Dated: February 21, 2026)

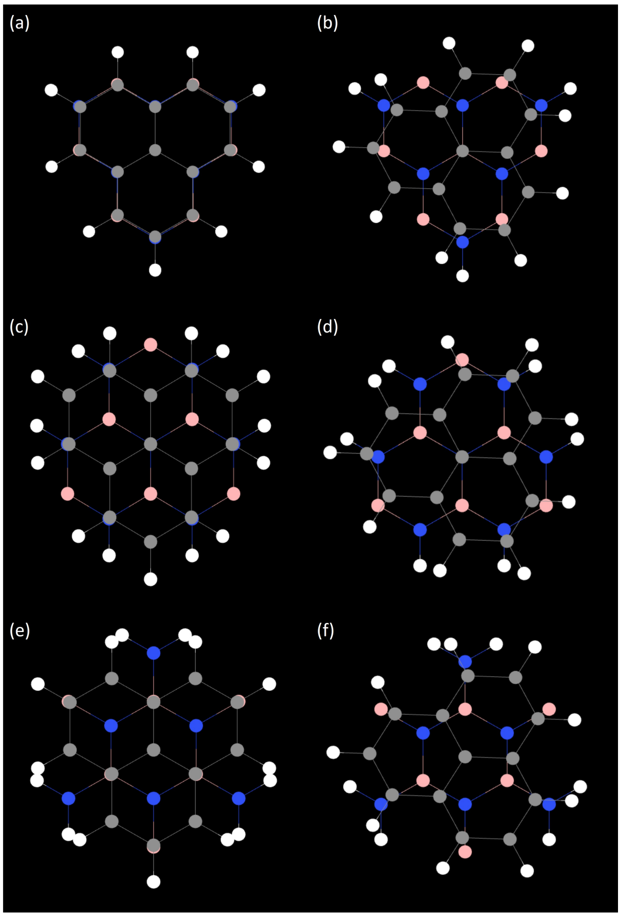

Figure S1: Schematic atomic structures of graphene/hBN bilayer heterostructures with different stacking configurations at 0° (unrotated) and 28.05° twist angles. (a)–(f) show the atomic arrangements of graphene/h-BN heterostructures for different stacking configurations, where (a),(b) denote AA stacking, (c),(d) denote AB stacking, and (e),(f) denote BA stacking. The gray spheres represent carbon atoms in graphene, the pink and blue spheres represent boron and nitrogen atoms in h-BN, respectively, and white spheres represent boundary atoms of the system.

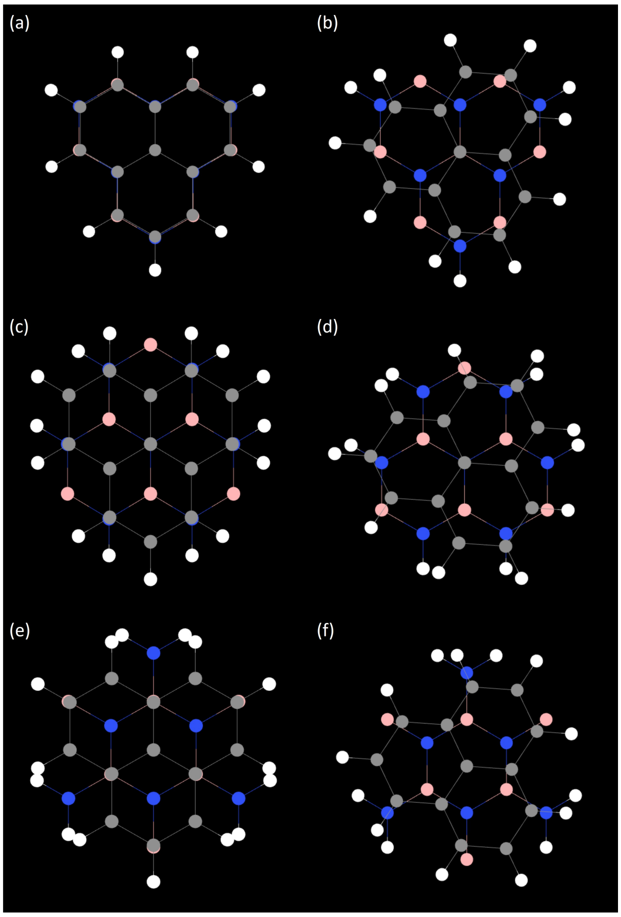

Figure S2: Schematic atomic structures of graphene/hBN bilayer heterostructures with different stacking configurations at 0° (unrotated) and 25.97° twist angles. (a)–(f) show the atomic arrangements of graphene/h-BN heterostructures for different stacking configurations, where (a),(b) denote AA stacking, (c),(d) denote AB stacking, and (e),(f) denote BA stacking. The gray spheres represent carbon atoms in graphene, the pink and blue spheres represent boron and nitrogen atoms in h-BN, respectively, and white spheres represent boundary atoms of the system.

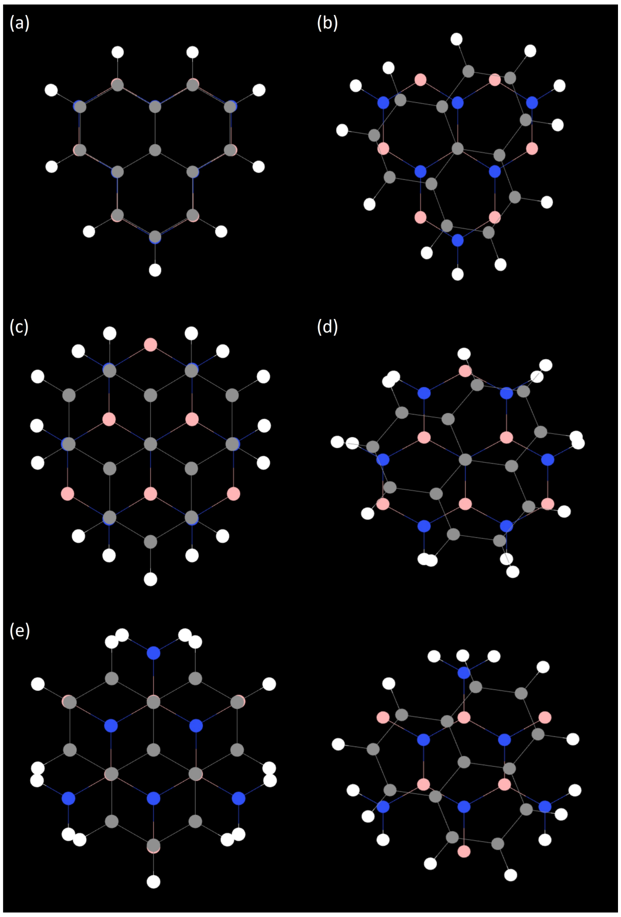

Figure S3: Schematic atomic structures of graphene/hBN bilayer heterostructures with different stacking configurations at 0° (unrotated) and 21.54° twist angles. (a)–(f) show the atomic arrangements of graphene/h-BN heterostructures for different stacking configurations, where (a),(b) denote AA stacking, (c),(d) denote AB stacking, and (e),(f) denote BA stacking. The gray spheres represent carbon atoms in graphene, the pink and blue spheres represent boron and nitrogen atoms in h-BN, respectively, and white spheres represent boundary atoms of the system.

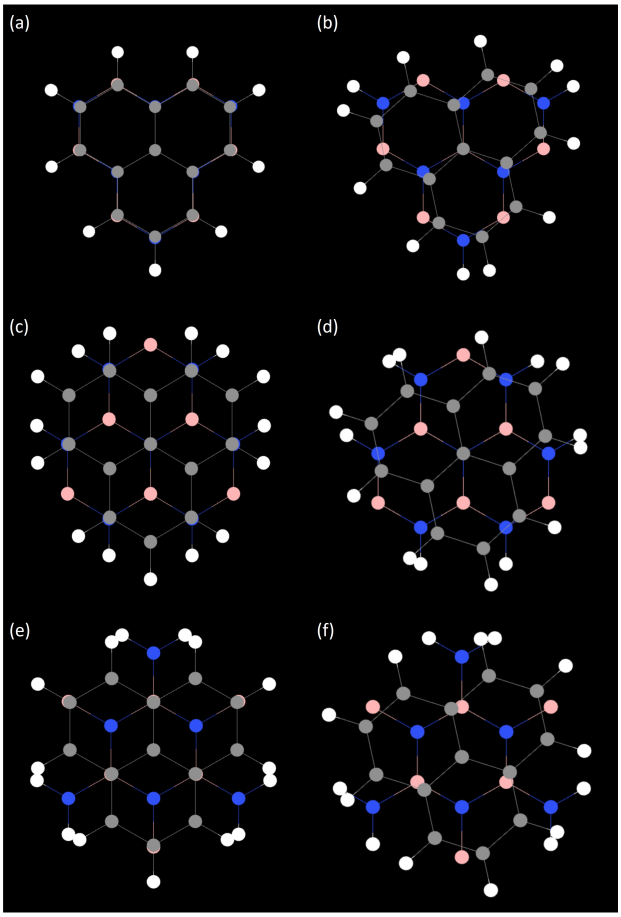

Figure S4: Schematic atomic structures of graphene/hBN bilayer heterostructures with different stacking configurations at 0° (unrotated) and 11.99° twist angles. (a)–(f) show the atomic arrangements of graphene/h-BN heterostructures for different stacking configurations, where (a),(b) denote AA stacking, (c),(d) denote AB stacking, and (e),(f) denote BA stacking. The gray spheres represent carbon atoms in graphene, the pink and blue spheres represent boron and nitrogen atoms in h-BN, respectively, and white spheres represent boundary atoms of the system.

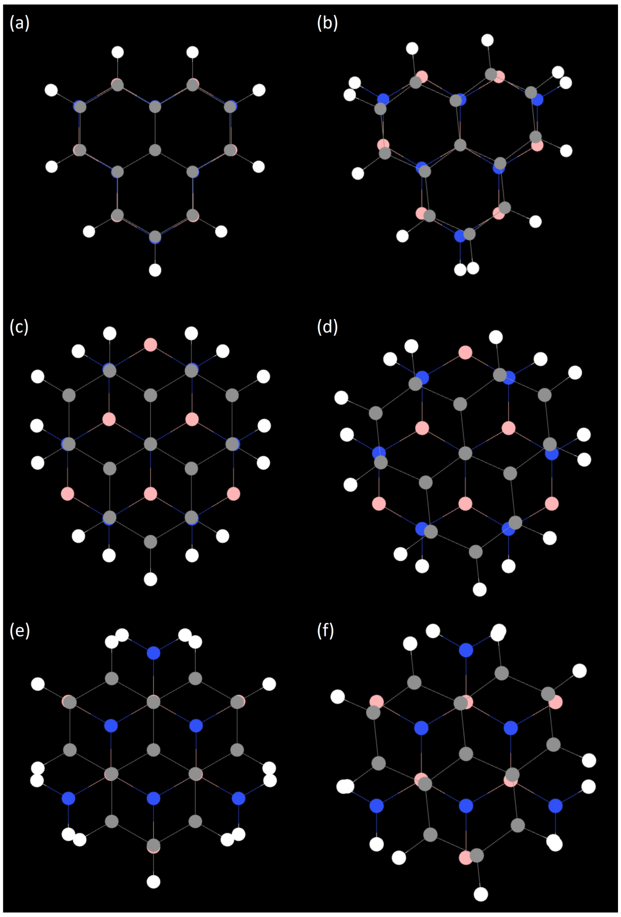

Figure S5: Schematic atomic structures of graphene/hBN bilayer heterostructures with different stacking configurations at 0° (unrotated) and 6.07° twist angles. (a)–(f) show the atomic arrangements of graphene/h-BN heterostructures for different stacking configurations, where (a),(b) denote AA stacking, (c),(d) denote AB stacking, and (e),(f) denote BA stacking. The gray spheres represent carbon atoms in graphene, the pink and blue spheres represent boron and nitrogen atoms in h-BN, respectively, and white spheres represent boundary atoms of the system.

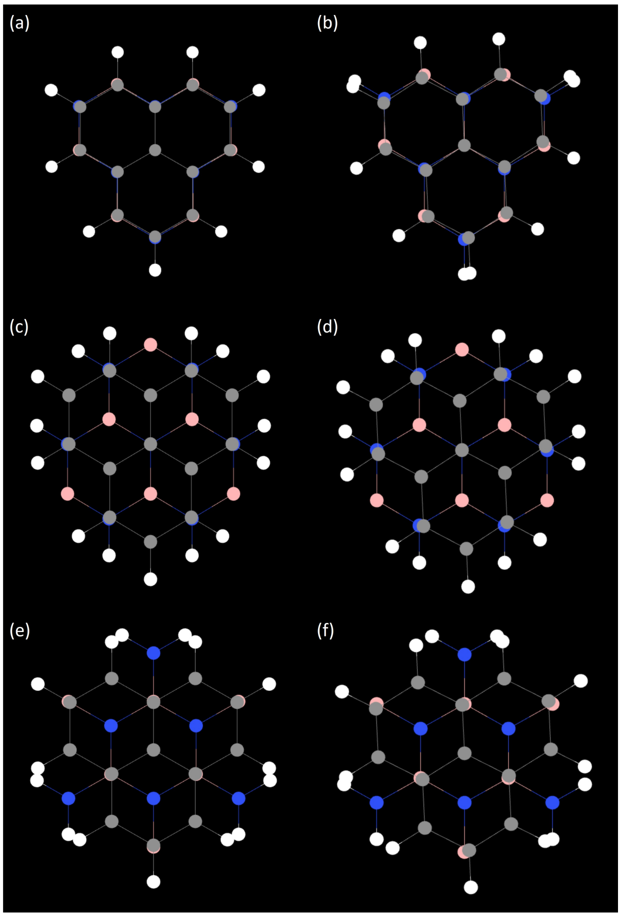

Figure S6: Schematic atomic structures of graphene/hBN bilayer heterostructures with different stacking configurations at 0° (unrotated) and 2.65° twist angles. (a)–(f) show the atomic arrangements of graphene/h-BN heterostructures for different stacking configurations, where (a),(b) denote AA stacking, (c),(d) denote AB stacking, and (e),(f) denote BA stacking. The gray spheres represent carbon atoms in graphene, the pink and blue spheres represent boron and nitrogen atoms in h-BN, respectively, and white spheres represent boundary atoms of the system.

Section II. Charge Stability Diagrams of TG/hBN Bilayer Heterostructures with Different Stacking Configurations

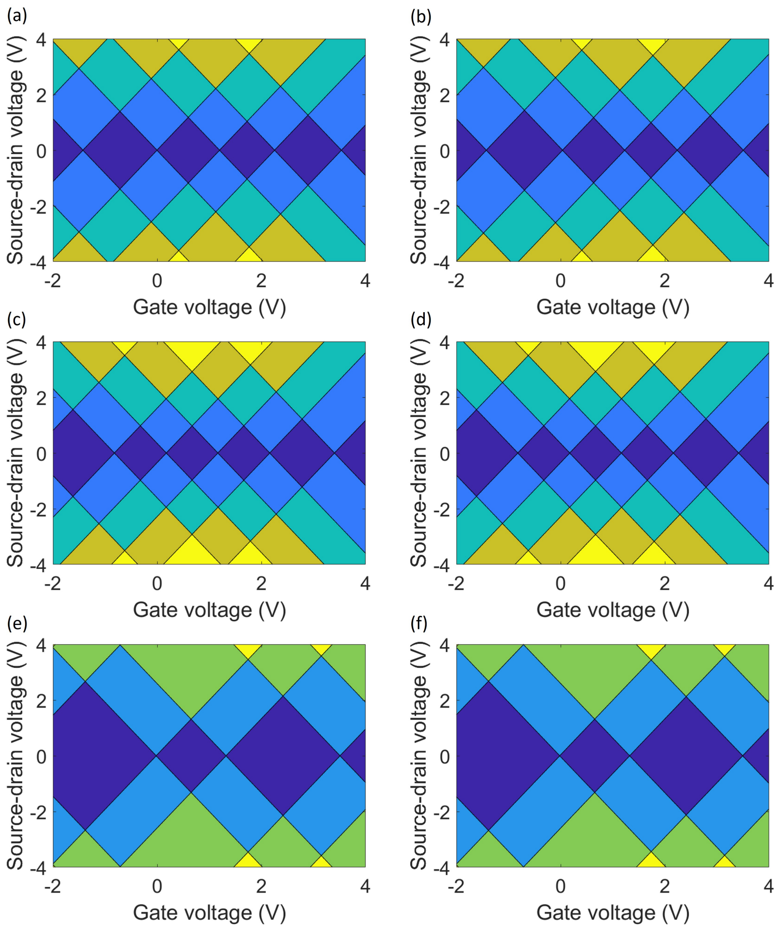

Figure S7: The left (right) column shows the charge stability diagram of the stacked charges AA, AB, BA from top to bottom without rotation (rotated 28.5°). The dark blue, blue, green, and yellow color schemes represent charge state numbers of 0, 1, 2, and 3, respectively.

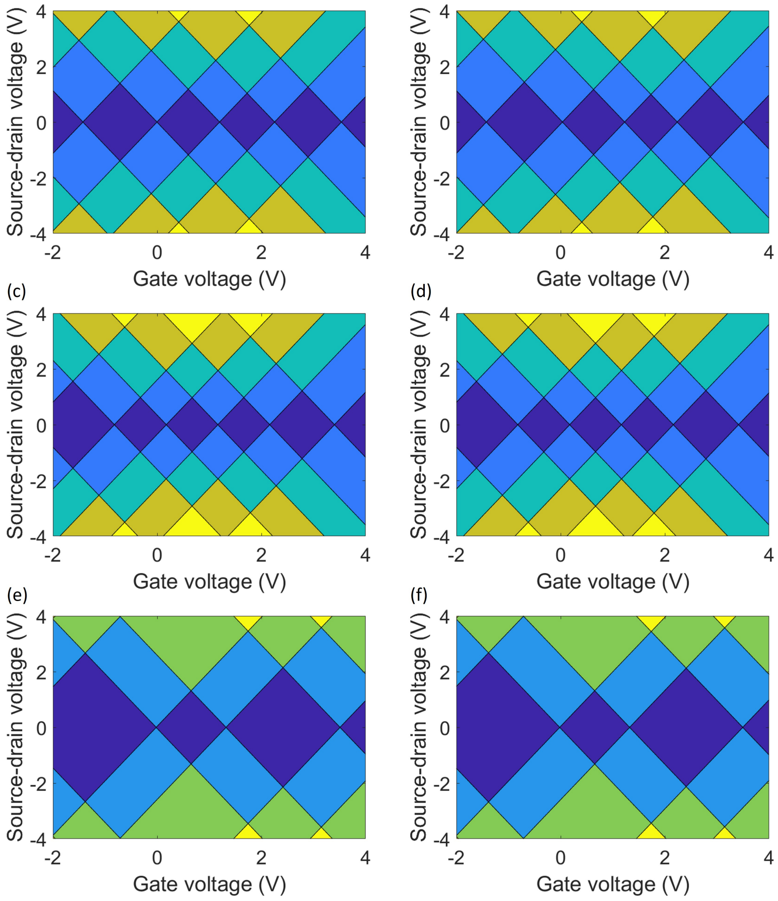

Figure S8: The left (right) column shows the charge stability diagram of the stacked charges AA, AB, BA from top to bottom without rotation (rotated 25.97°). The dark blue, blue, green, and yellow color schemes represent charge state numbers of 0, 1, 2, and 3, respectively.

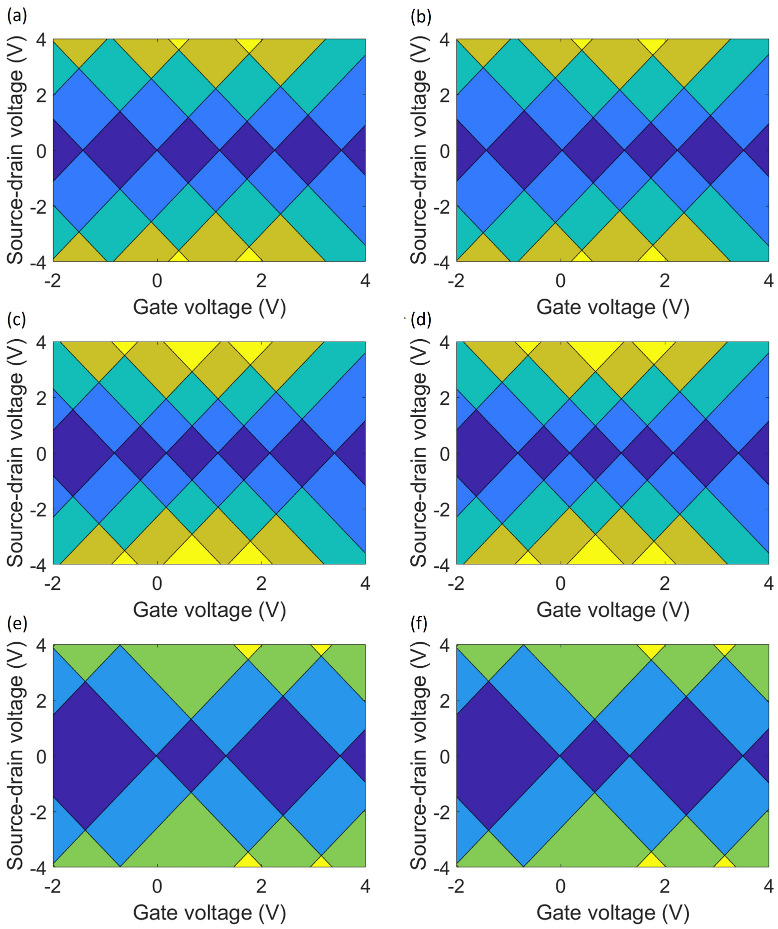

Figure S9: The left (right) column shows the charge stability diagram of the stacked charges AA, AB, BA from top to bottom without rotation (rotated 21.54°). The dark blue, blue, green, and yellow color schemes represent charge state numbers of 0, 1, 2, and 3, respectively.

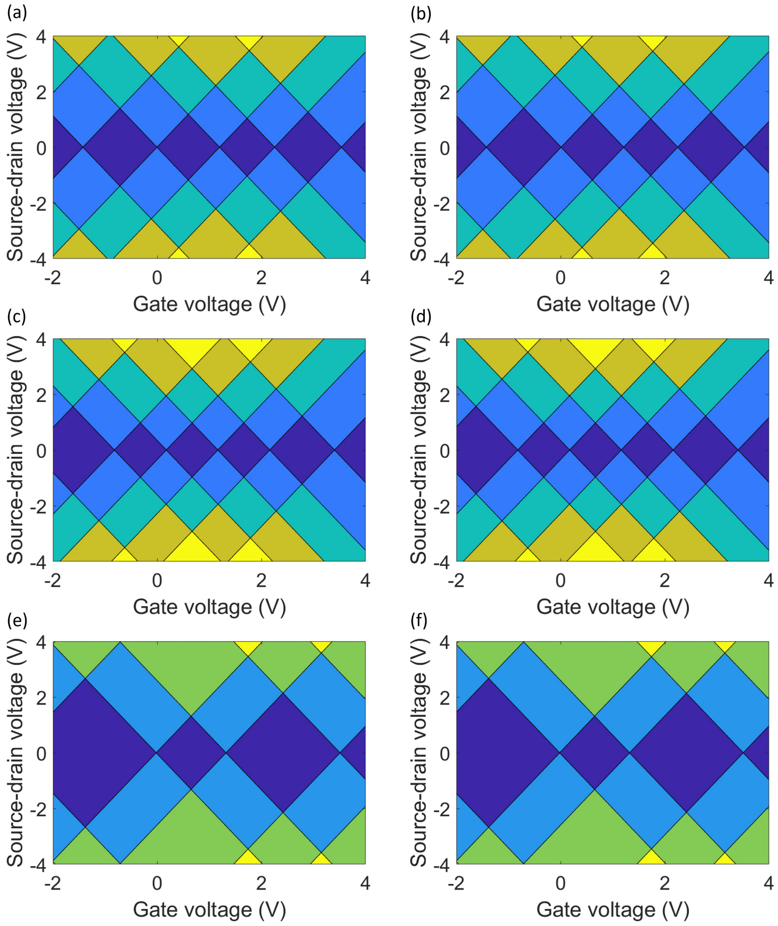

Figure S10: The left (right) column shows the charge stability diagram of the stacked charges AA, AB, BA from top to bottom without rotation (rotated 11.99°). The dark blue, blue, green, and yellow color schemes represent charge state numbers of 0, 1, 2, and 3, respectively.

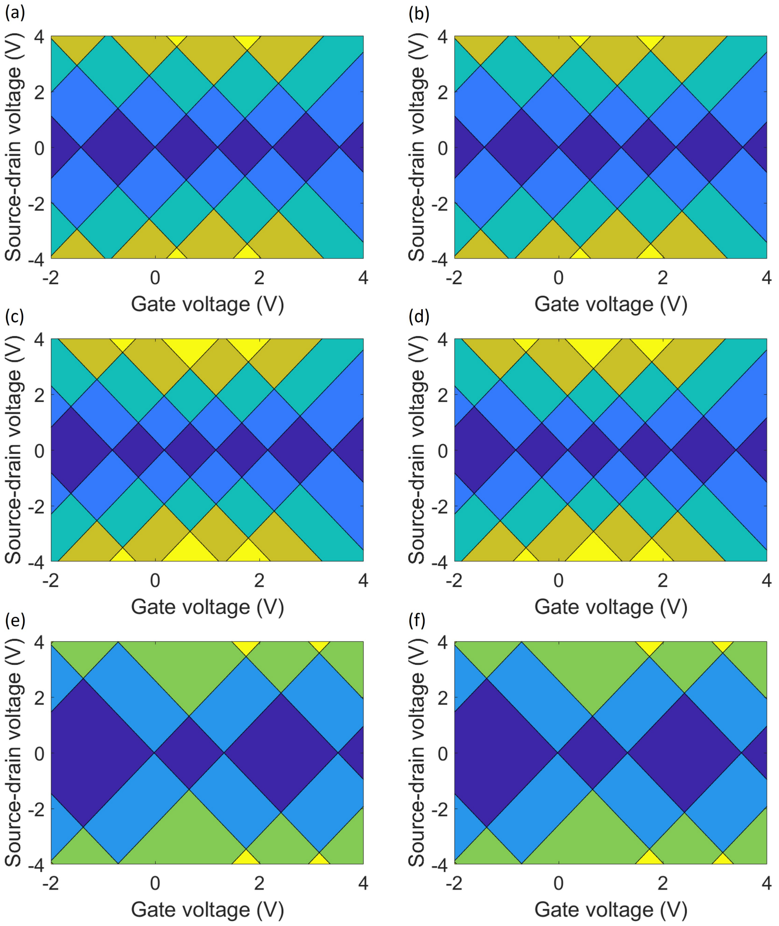

Figure S11: The left (right) column shows the charge stability diagram of the stacked charges AA, AB, BA from top to bottom without rotation (rotated 6.07°). The dark blue, blue, green, and yellow color schemes represent charge state numbers of 0, 1, 2, and 3, respectively.

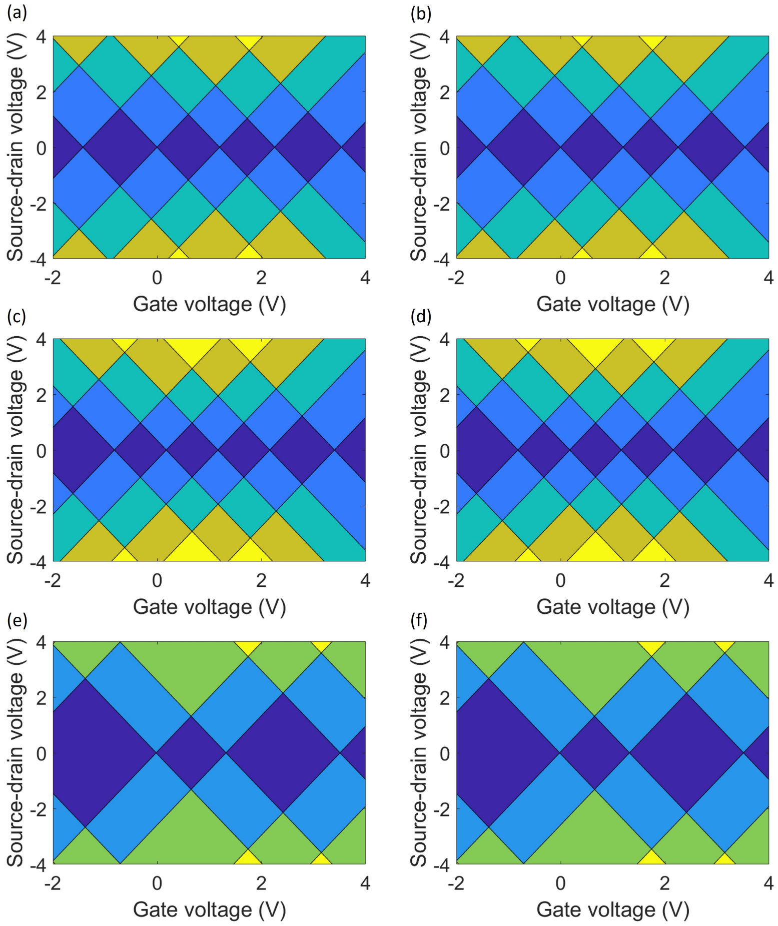

Figure S12: The left (right) column shows the charge stability diagram of the stacked charges AA, AB, BA from top to bottom without rotation (rotated 2.65°). The dark blue, blue, green, and yellow color schemes represent charge state numbers of 0, 1, 2, and 3, respectively.

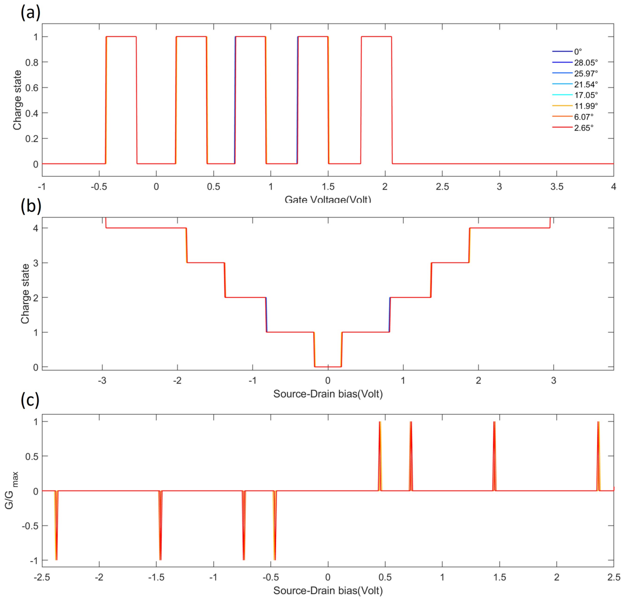

Figure S13: A detailed study of the charge stability plots for the gate t = b configuration of the AA stacking with a radius of r2. (a) shows the line scan along the gate voltage (source-drain bias equal to 0); (b) shows the line scan along the source-drain bias at the central diamond maximum (gate voltage = 3.208 V); (c) depicts the normalized differential conductance relative to the source-drain bias in (b).

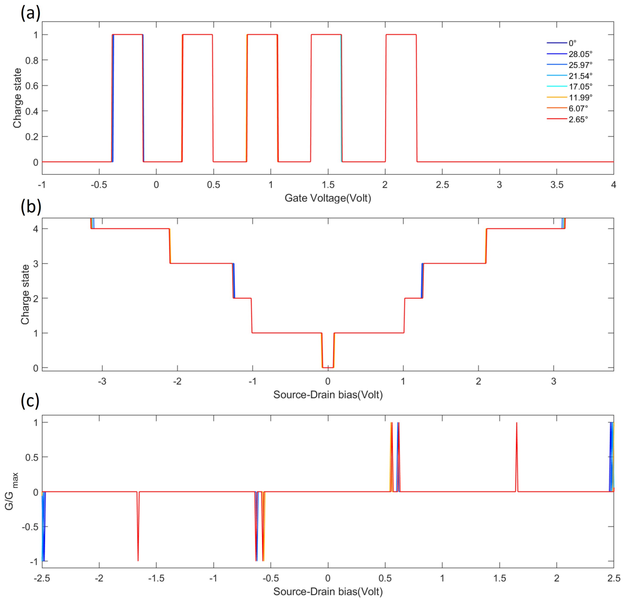

Figure S14: A detailed study of the charge stability plots for the gate t = b configuration of the AB stacking with a radius of r2. (a) shows the line scan along the gate voltage (source-drain bias equal to 0); (b) shows the line scan along the source-drain bias at the central diamond maximum (gate voltage = 3.208 V); (c) depicts the normalized differential conductance relative to the source-drain bias in (b).

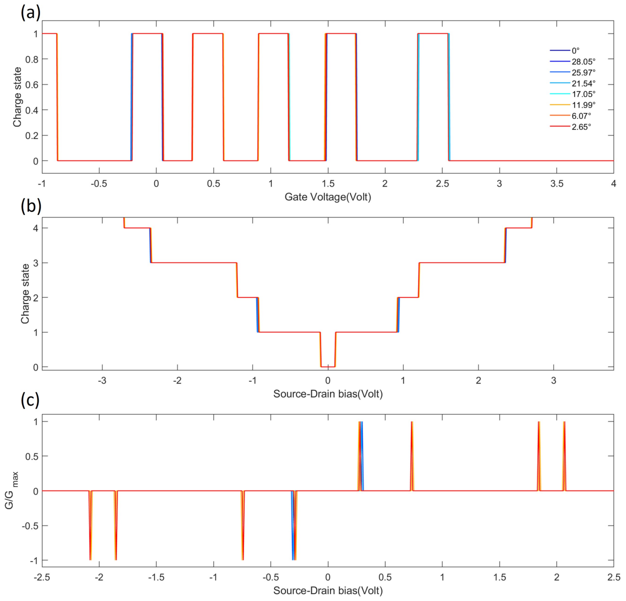

Figure S15: A detailed study of the charge stability plots for the gate t = b configuration of the BA stacking with a radius of r2. (a) shows the line scan along the gate voltage (source-drain bias equal to 0); (b) shows the line scan along the source-drain bias at the central diamond maximum (gate voltage = 3.208 V); (c) depicts the normalized differential conductance relative to the source-drain bias in (b).

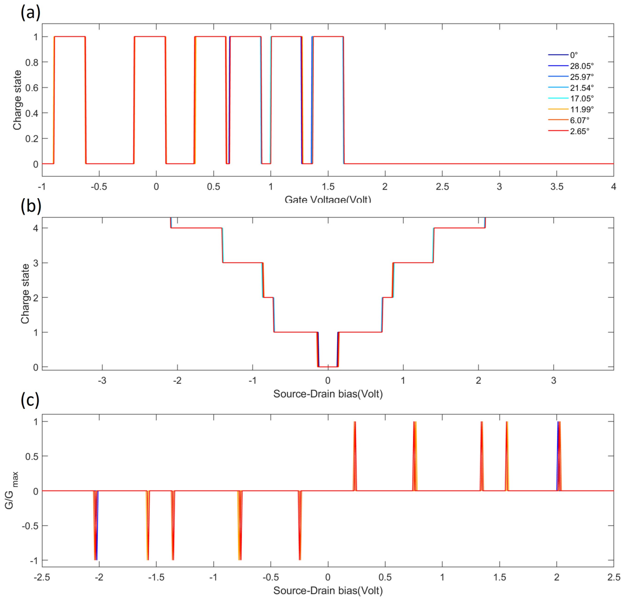

Figure S16: A detailed study of the charge stability plots for the gate t = b configuration of the AA stacking with a radius of r3. (a) shows the line scan along the gate voltage (source-drain bias equal to 0); (b) shows the line scan along the source-drain bias at the central diamond maximum (gate voltage = 3.208 V); (c) depicts the normalized differential conductance relative to the source-drain bias in (b).

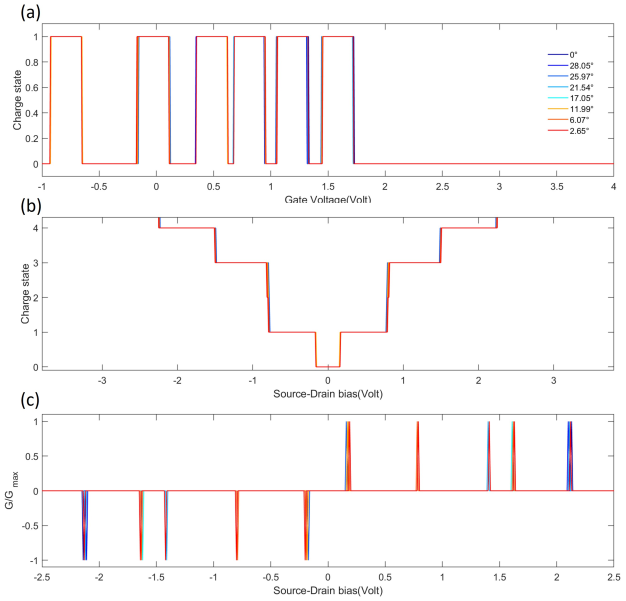

Figure S17: A detailed study of the charge stability plots for the gate t = b configuration of the AB stacking with a radius of r3. (a) shows the line scan along the gate voltage (source-drain bias equal to 0); (b) shows the line scan along the source-drain bias at the central diamond maximum (gate voltage = 3.208 V); (c) depicts the normalized differential conductance relative to the source-drain bias in (b).

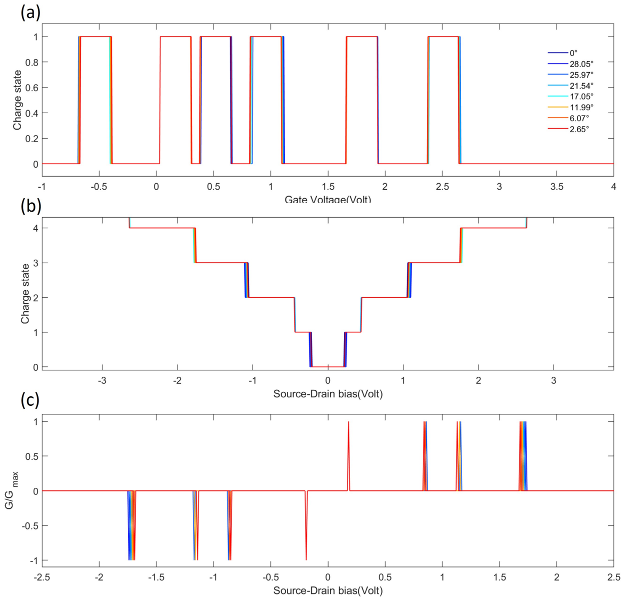

Figure S18: A detailed study of the charge stability plots for the gate t = b configuration of the BA stacking with a radius of r3. (a) shows the line scan along the gate voltage (source-drain bias equal to 0); (b) shows the line scan along the source-drain bias at the central diamond maximum (gate voltage = 3.208 V); (c) depicts the normalized differential conductance relative to the source-drain bias in (b).

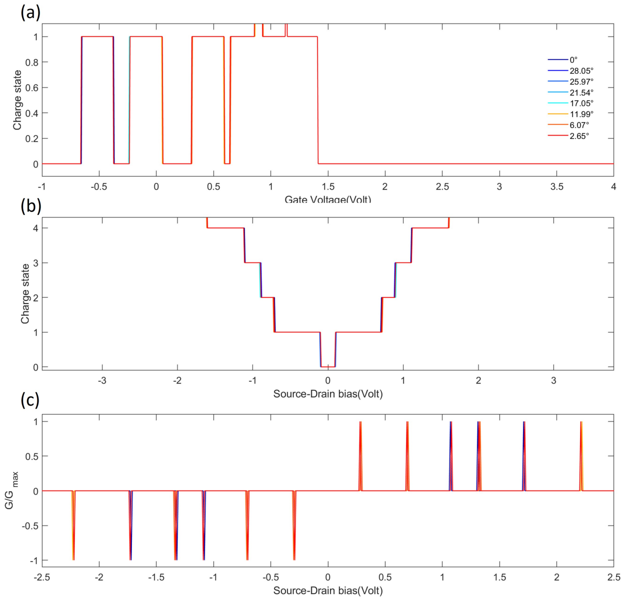

Figure S19: A detailed study of the charge stability plots for the gate t = b configuration of the AA stacking with a radius of r3. (a) shows the line scan along the gate voltage (source-drain bias equal to 0); (b) shows the line scan along the source-drain bias at the central diamond maximum (gate voltage = 3.208 V); (c) depicts the normalized differential conductance relative to the source-drain bias in (b).

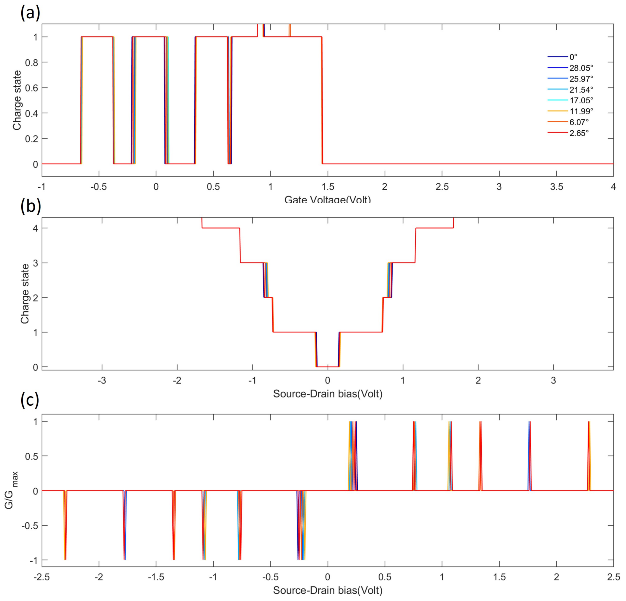

Figure S20: A detailed study of the charge stability plots for the gate t = b configuration of the AB stacking with a radius of r3. (a) shows the line scan along the gate voltage (source-drain bias equal to 0); (b) shows the line scan along the source-drain bias at the central diamond maximum (gate voltage = 3.208 V); (c) depicts the normalized differential conductance relative to the source-drain bias in (b).

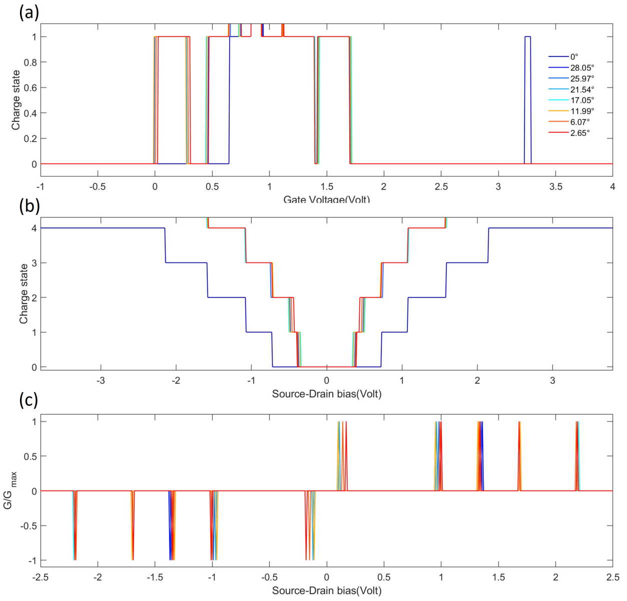

Figure S21: A detailed study of the charge stability plots for the gate t = b configuration of the BA stacking with a radius of r3. (a) shows the line scan along the gate voltage (source-drain bias equal to 0); (b) shows the line scan along the source-drain bias at the central diamond maximum (gate voltage = 3.208 V); (c) depicts the normalized differential conductance relative to the source-drain bias in (b).

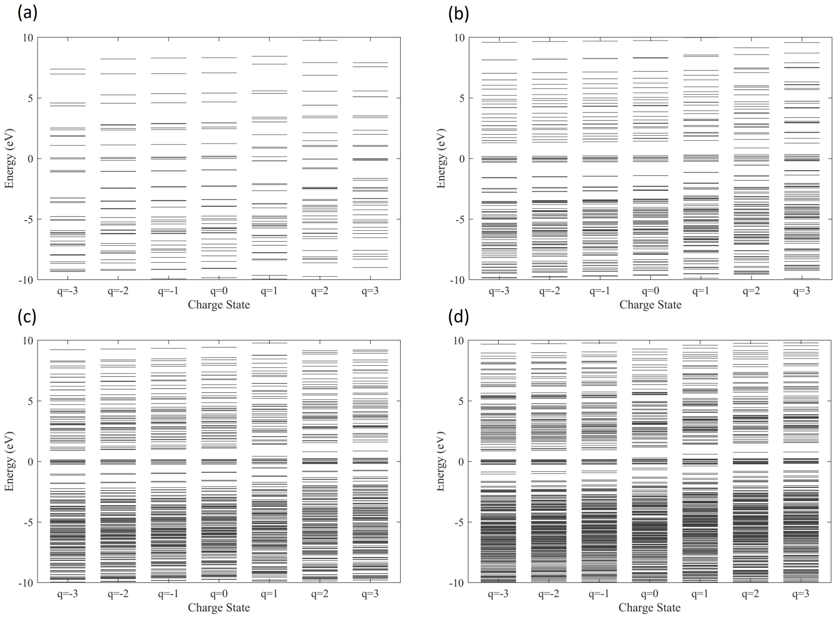

Figure S22: The molecular energy spectra of seven charge states of unrotated AA-stacked TG/hBN bilayer heterojunction quantum dots with four different radii (r1,r2,r3,r4)

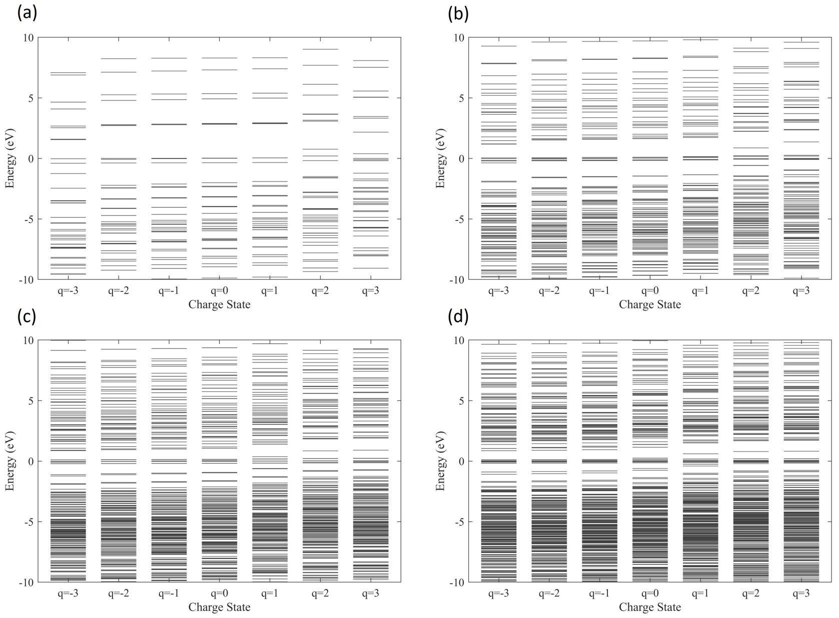

Figure S23: The molecular energy spectra of seven charge states of unrotated AB-stacked TG/hBN bilayer heterojunction quantum dots with four different radii (r1,r2,r3,r4)

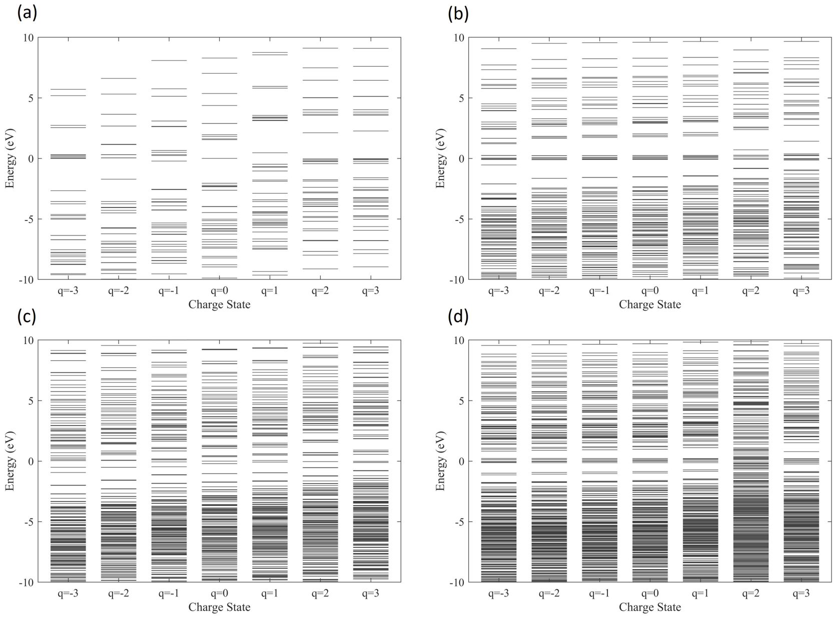

Figure S24: The molecular energy spectra of seven charge states of unrotated BA-stacked TG/hBN bilayer heterojunction quantum dots with four different radii (r1,r2,r3,r4)

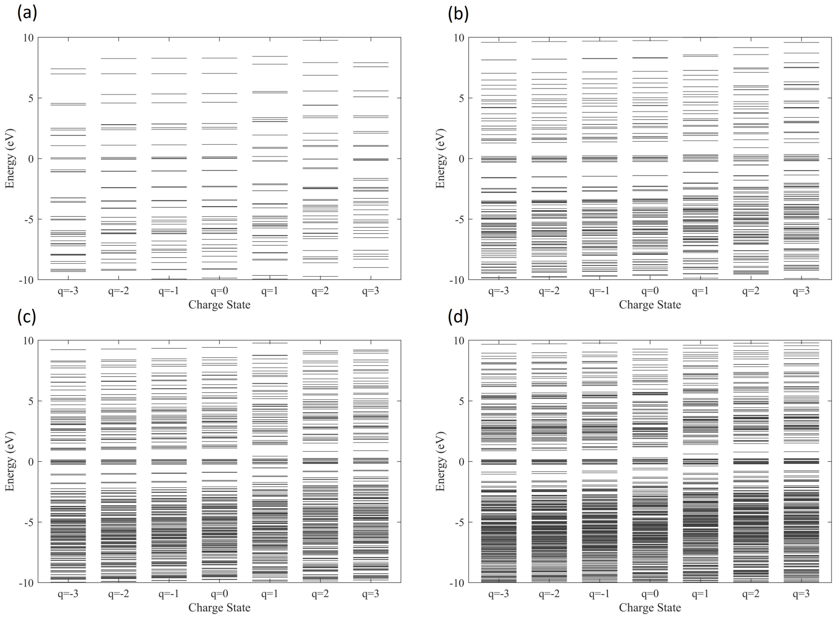

Figure S25: The molecular energy spectra of seven charge states of 28.05° rotated AA-stacked TG/hBN bilayer heterojunction quantum dots with four different radii (r1,r2,r3,r4)

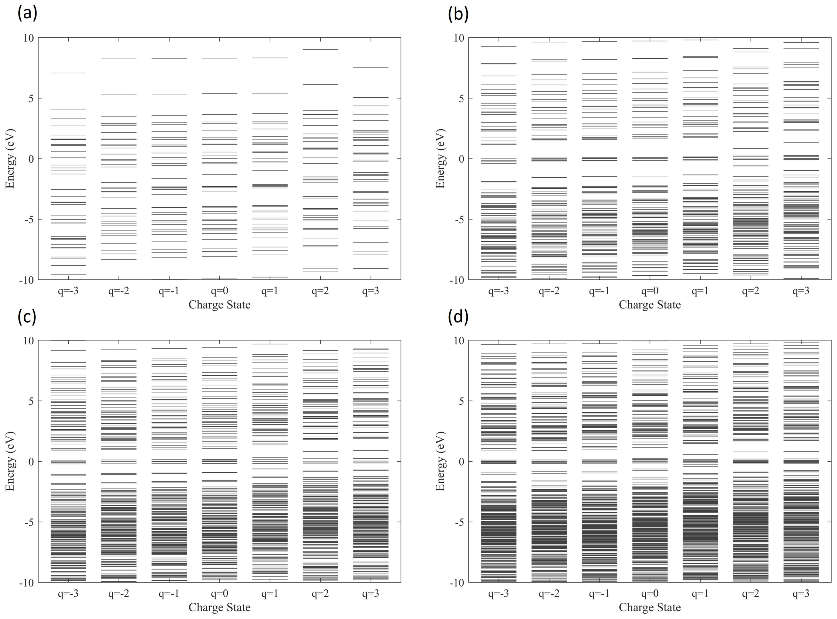

Figure S26: The molecular energy spectra of seven charge states of 28.05° rotated AB-stacked TG/hBN bilayer heterojunction quantum dots with four different radii (r1,r2,r3,r4)

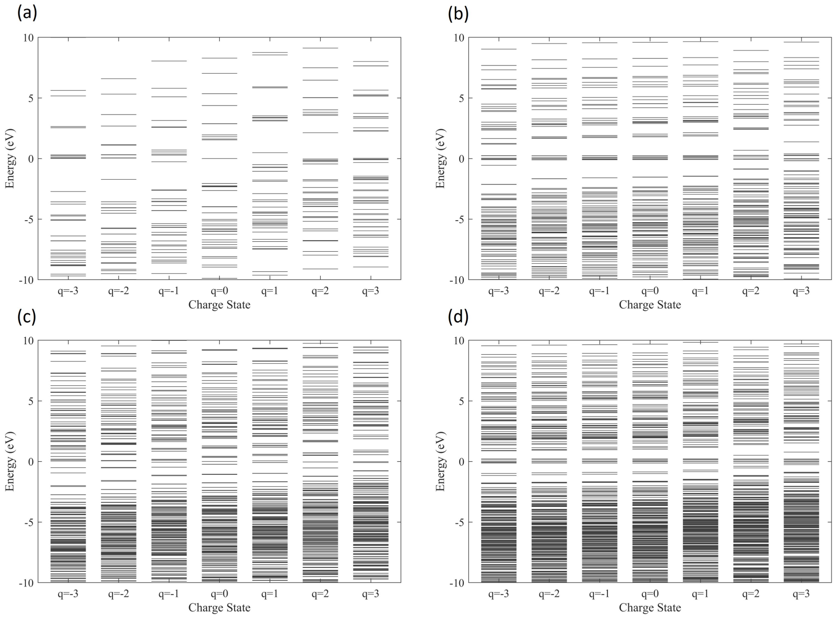

Figure S27: The molecular energy spectra of seven charge states of 28.05° rotated BA-stacked TG/hBN bilayer heterojunction quantum dots with four different radii (r1,r2,r3,r4)

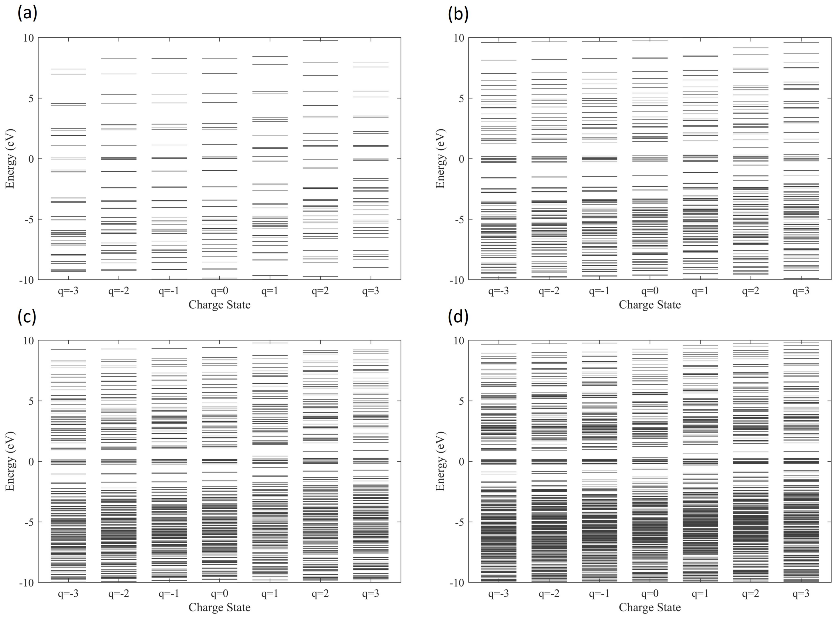

Figure S28: The molecular energy spectra of seven charge states of 25.97° rotated AA-stacked TG/hBN bilayer heterojunction quantum dots with four different radii (r1,r2,r3,r4)

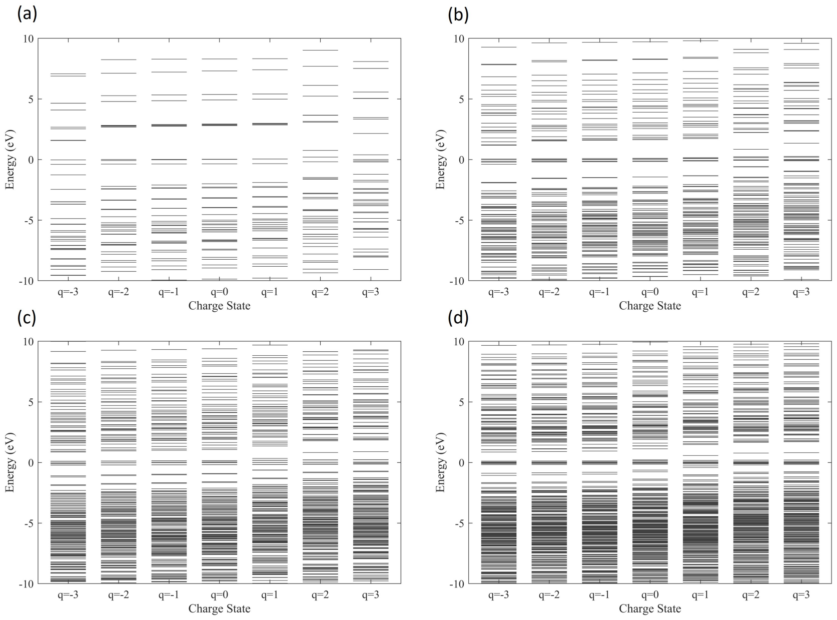

Figure S29: The molecular energy spectra of seven charge states of 25.97° rotated AB-stacked TG/hBN bilayer heterojunction quantum dots with four different radii (r1,r2,r3,r4)

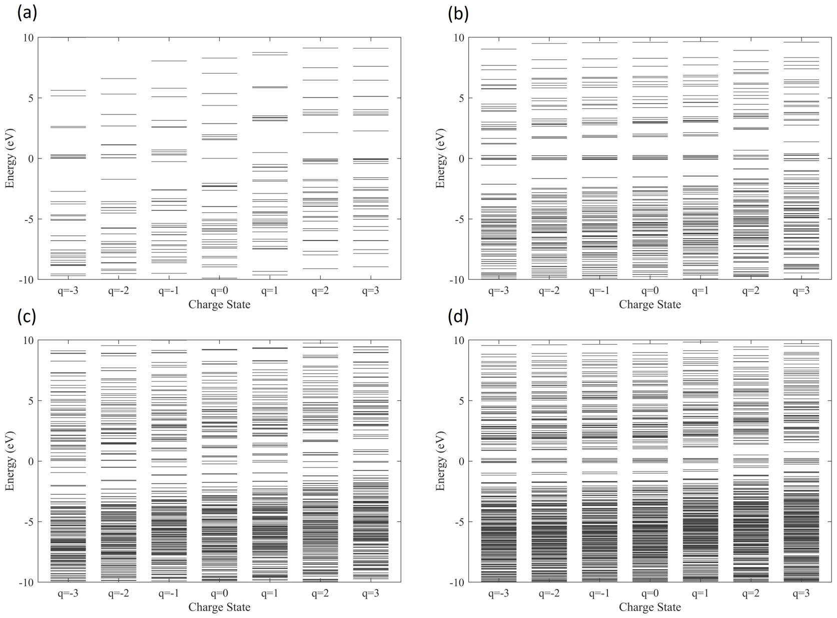

Figure S30: The molecular energy spectra of seven charge states of 25.97° rotated BA-stacked TG/hBN bilayer heterojunction quantum dots with four different radii (r1,r2,r3,r4)

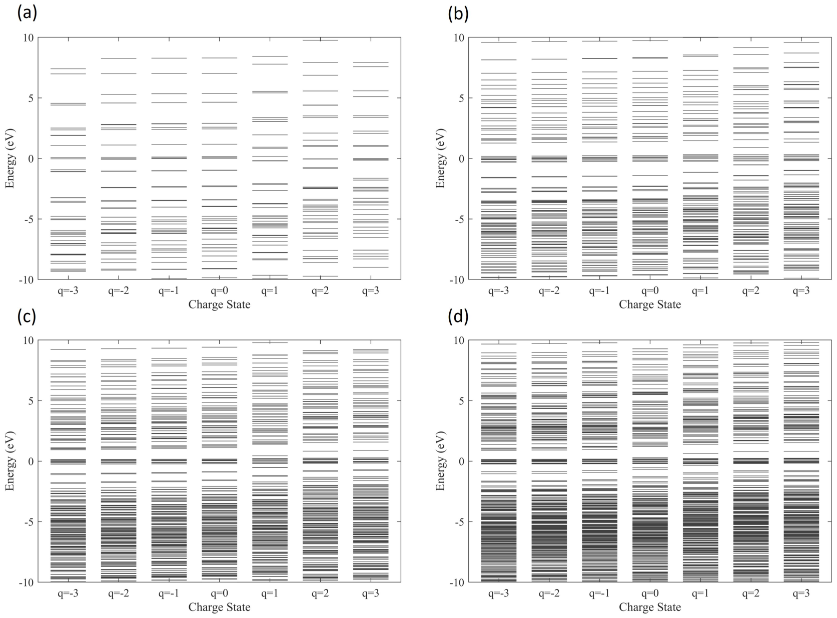

Figure S31: The molecular energy spectra of seven charge states of 21.54° rotated AA-stacked TG/hBN bilayer heterojunction quantum dots with four different radii (r1,r2,r3,r4)

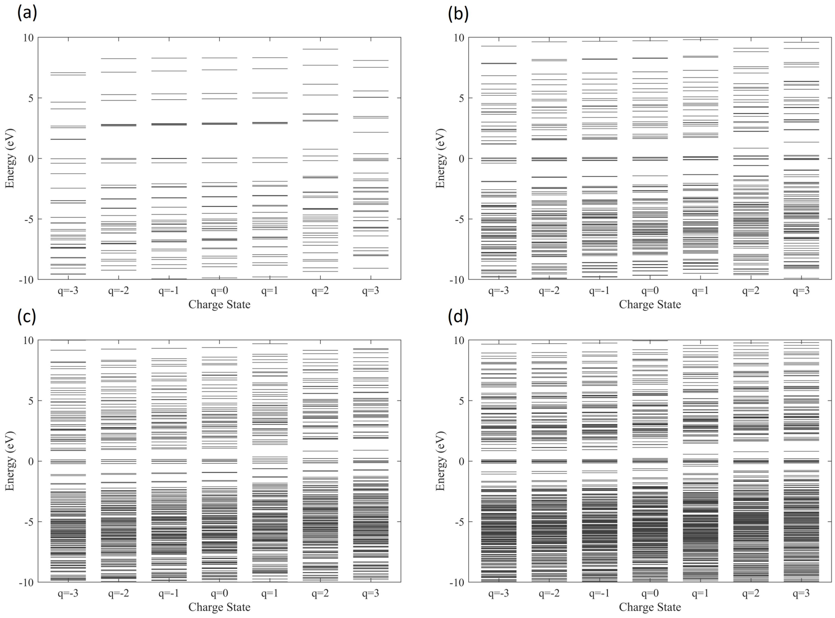

Figure S32: The molecular energy spectra of seven charge states of 21.54° rotated AB-stacked TG/hBN bilayer heterojunction quantum dots with four different radii (r1,r2,r3,r4)

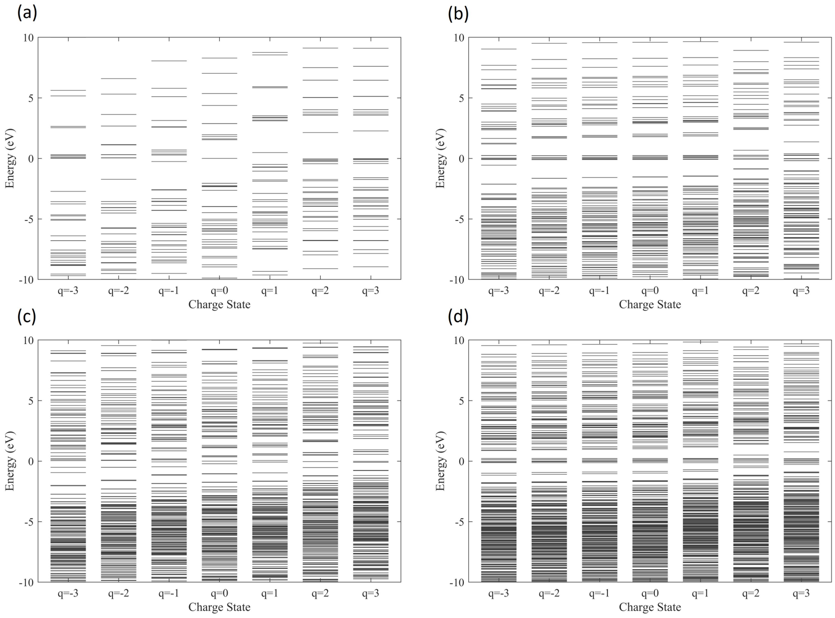

Figure S33: The molecular energy spectra of seven charge states of 21.54° rotated BA-stacked TG/hBN bilayer heterojunction quantum dots with four different radii (r1,r2,r3,r4)

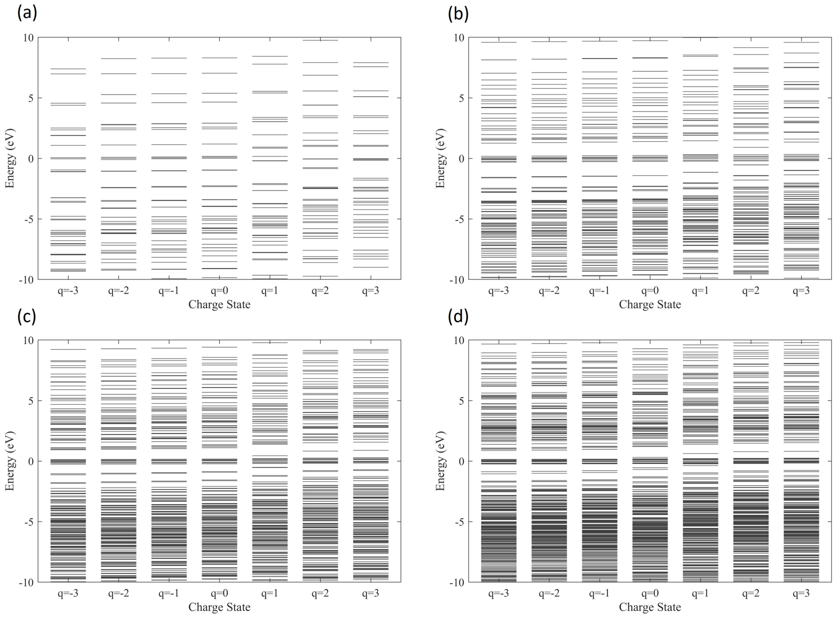

Figure S34: The molecular energy spectra of seven charge states of 17.05° rotated AA-stacked TG/hBN bilayer heterojunction quantum dots with four different radii (r1,r2,r3,r4)

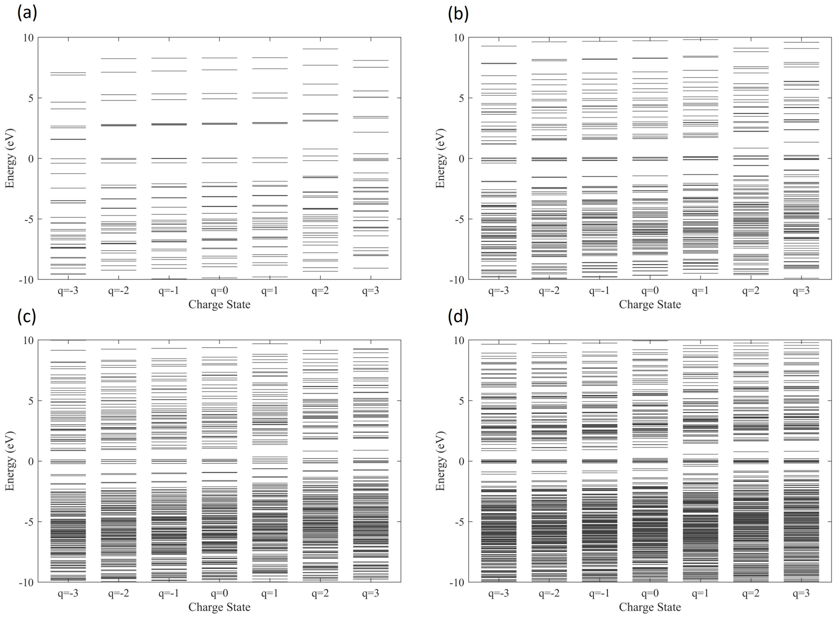

Figure S35: The molecular energy spectra of seven charge states of 17.05° rotated AB-stacked TG/hBN bilayer heterojunction quantum dots with four different radii (r1,r2,r3,r4)

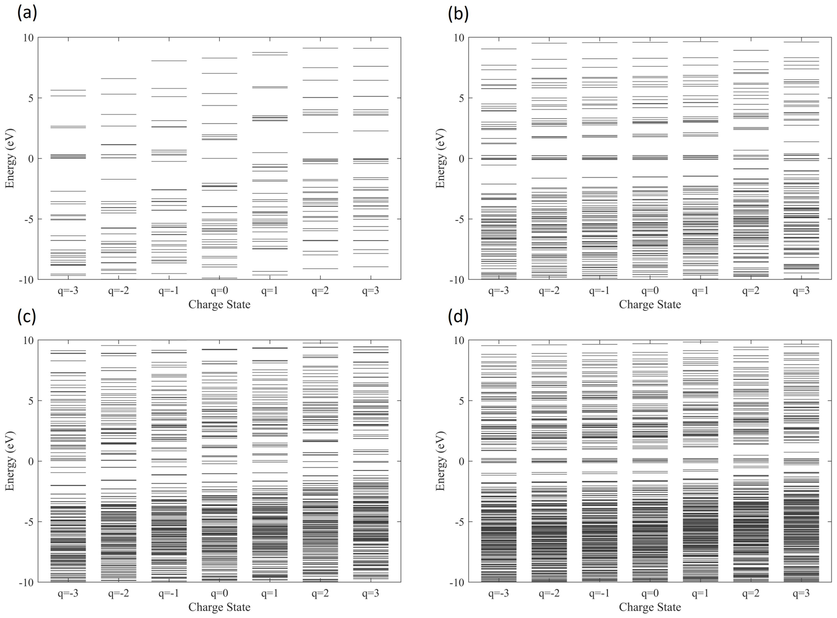

Figure S36: The molecular energy spectra of seven charge states of 17.05° rotated BA-stacked TG/hBN bilayer heterojunction quantum dots with four different radii (r1,r2,r3,r4)

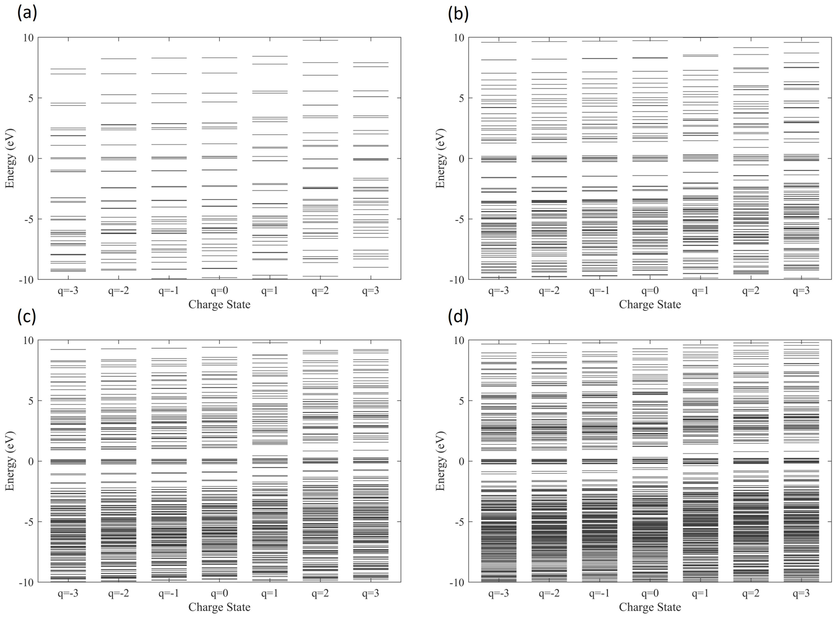

Figure S37: The molecular energy spectra of seven charge states of 11.99° rotated AA-stacked TG/hBN bilayer heterojunction quantum dots with four different radii (r1,r2,r3,r4)

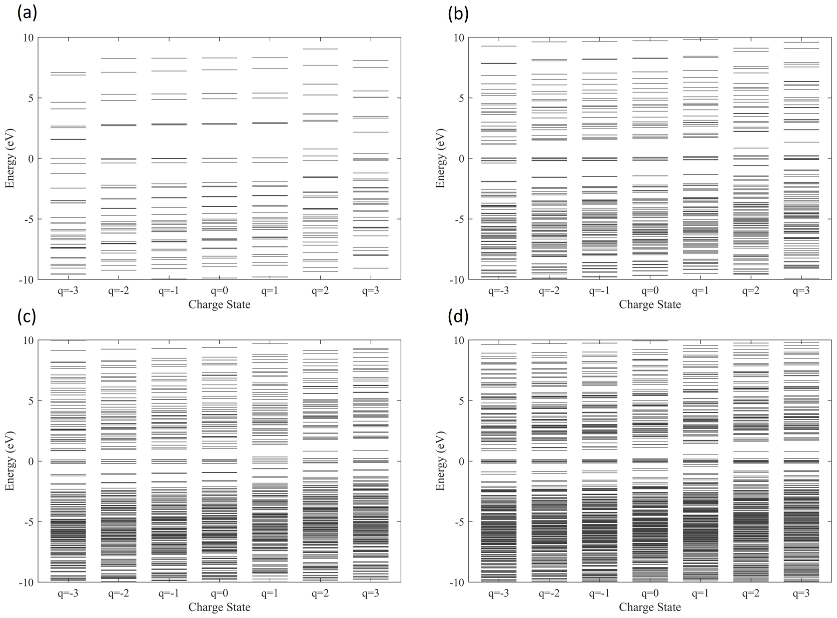

Figure S38: The molecular energy spectra of seven charge states of 11.99° rotated AB-stacked TG/hBN bilayer heterojunction quantum dots with four different radii (r1,r2,r3,r4)

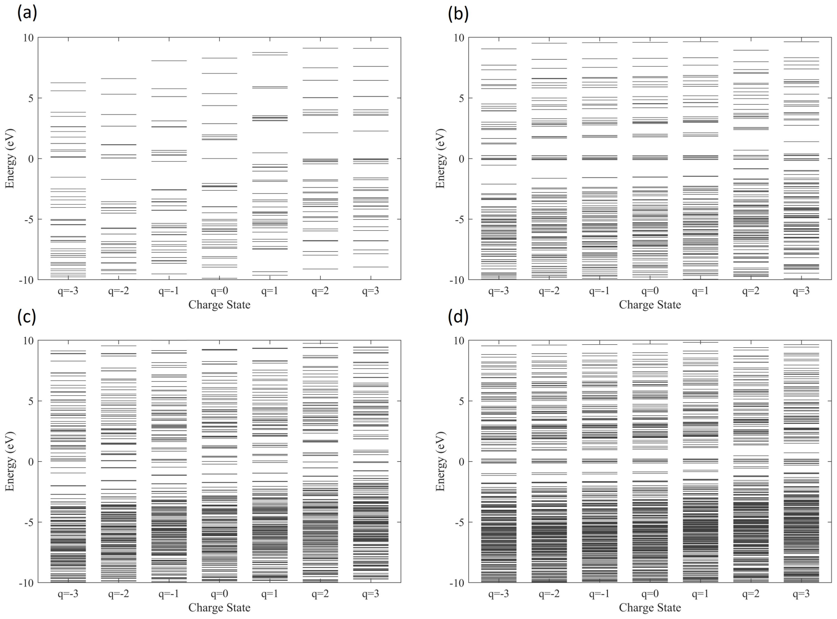

Figure S39: The molecular energy spectra of seven charge states of 11.99° rotated BA-stacked TG/hBN bilayer heterojunction quantum dots with four different radii (r1,r2,r3,r4)

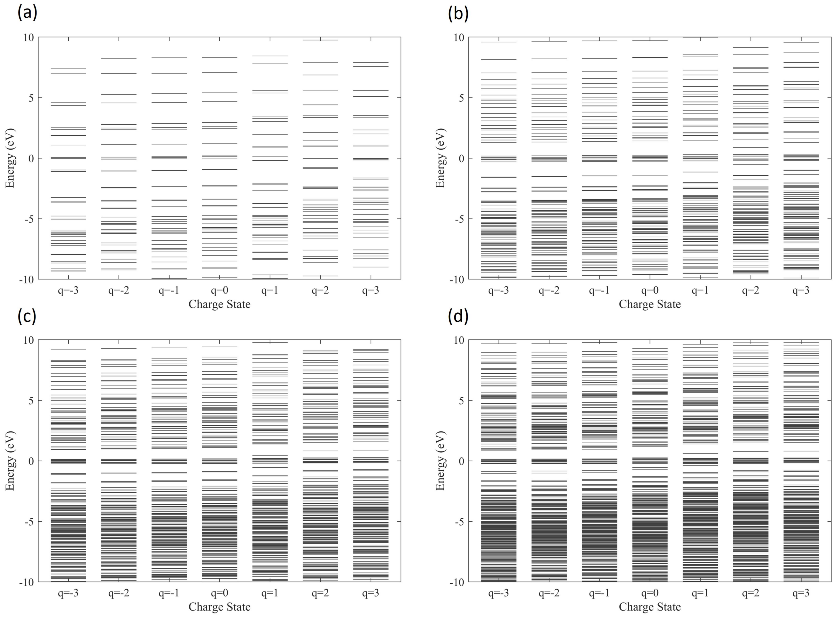

Figure S40: The molecular energy spectra of seven charge states of 6.07° rotated AA-stacked TG/hBN bilayer heterojunction quantum dots with four different radii (r1,r2,r3,r4)

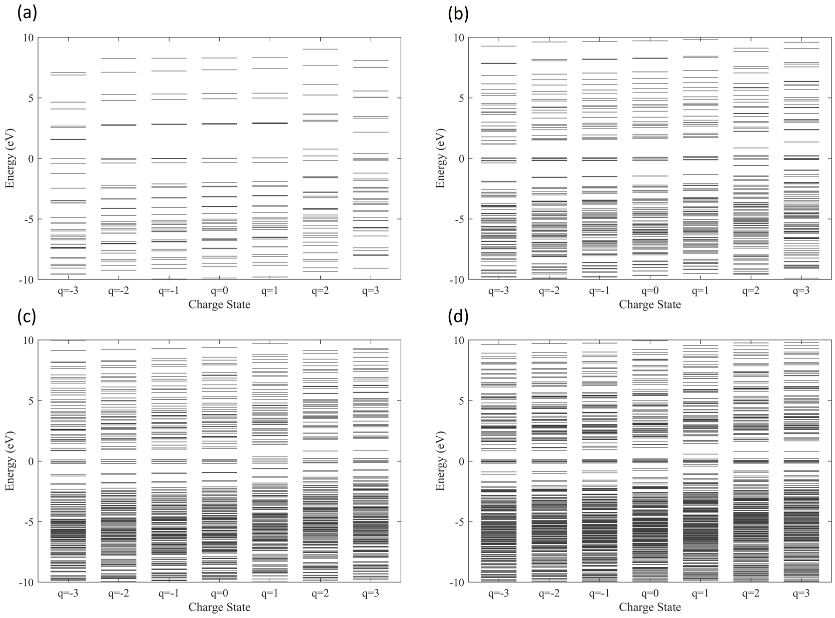

Figure S41: The molecular energy spectra of seven charge states of 6.07° rotated AB-stacked TG/hBN bilayer heterojunction quantum dots with four different radii (r1,r2,r3,r4)

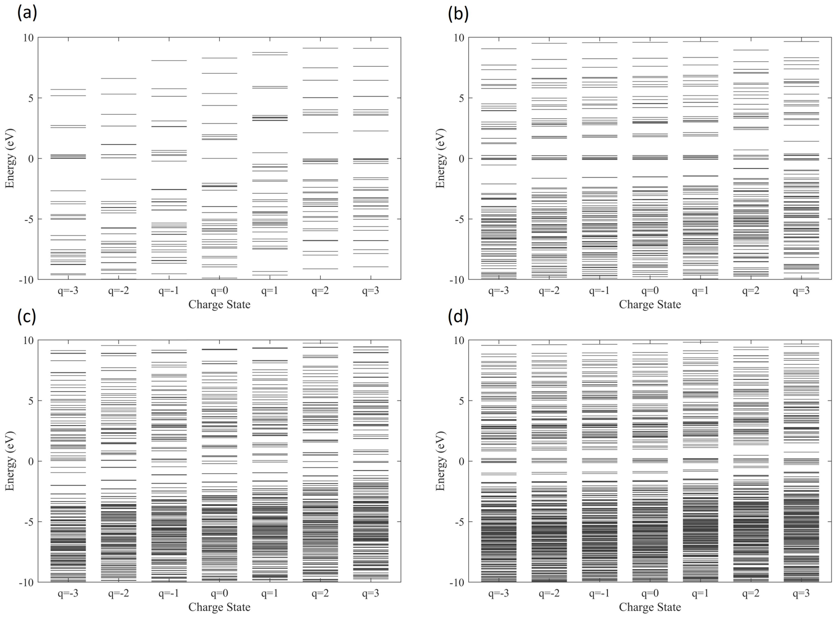

Figure S42: The molecular energy spectra of seven charge states of 6.07° rotated BA-stacked TG/hBN bilayer heterojunction quantum dots with four different radii (r1,r2,r3,r4)

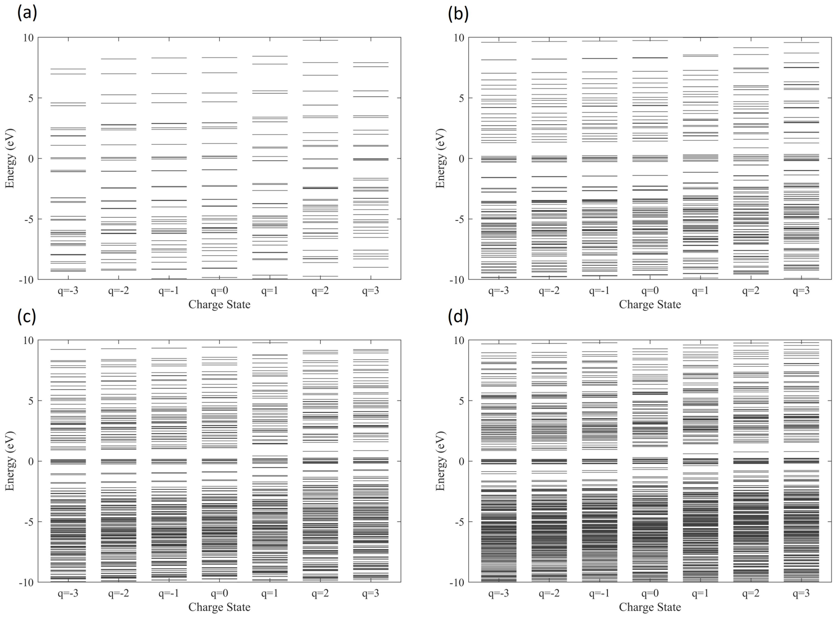

Figure S43: The molecular energy spectra of seven charge states of 2.65° rotated AA-stacked TG/hBN bilayer heterojunction quantum dots with four different radii (r1,r2,r3,r4)

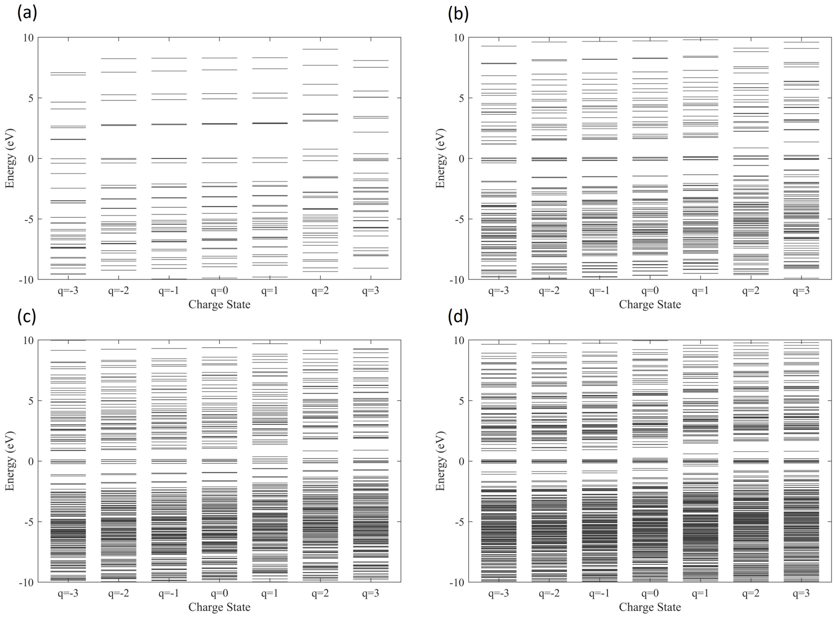

Figure S44: The molecular energy spectra of seven charge states of 2.65° rotated AB-stacked TG/hBN bilayer heterojunction quantum dots with four different radii (r1,r2,r3,r4)

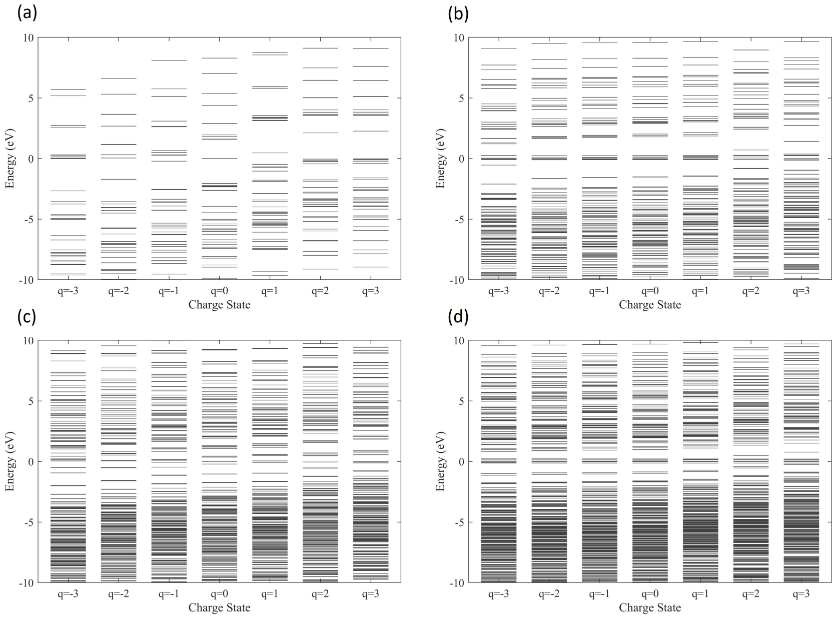

Figure S45: The molecular energy spectra of seven charge states of 2.65° rotated BA-stacked TG/hBN bilayer heterojunction quantum dots with four different radii (r1,r2,r3,r4)

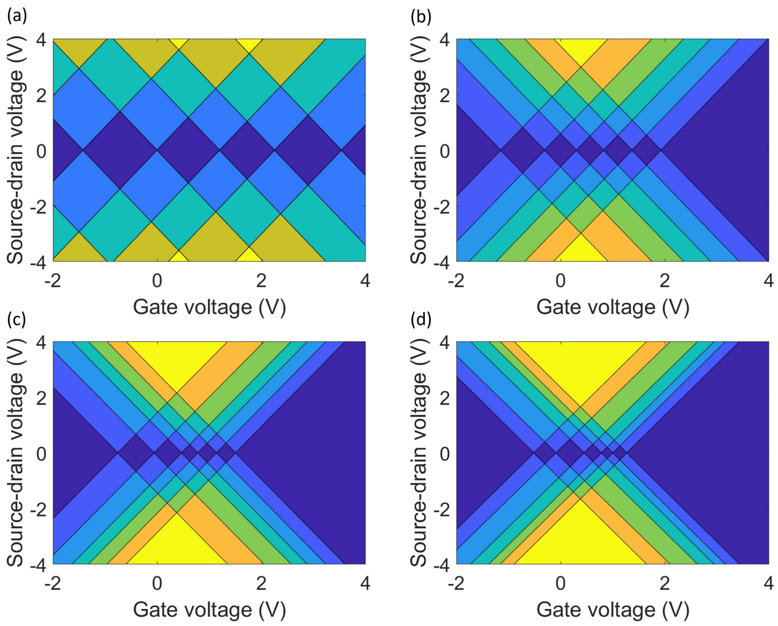

Figure S46: The charge stability plots of unrotated AA-stacked TG/hBN bilayer heterojunction quantum dots with four different radii (r1,r2,r3,r4).The dark blue, blue, green, and yellow color schemes represent charge state numbers of 0, 1, 2, and 3, respectively.

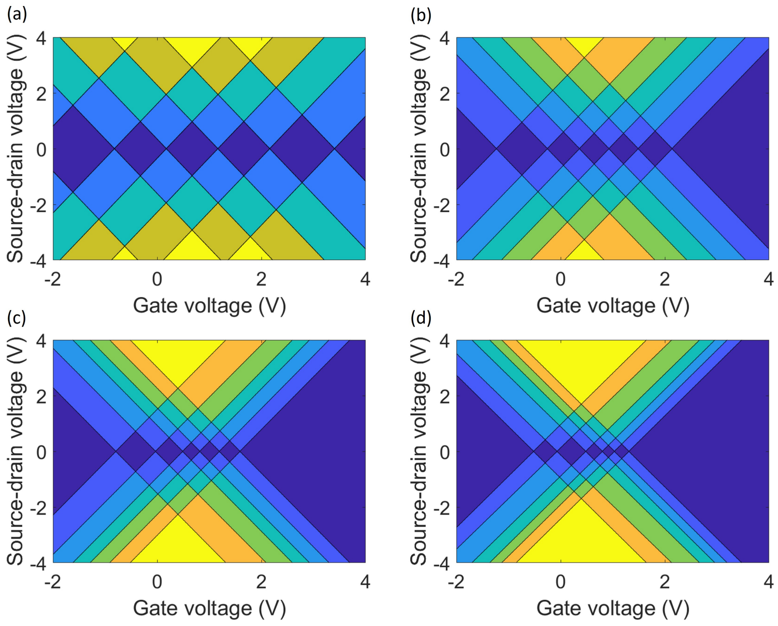

Figure S47: The charge stability plots of unrotated AB-stacked TG/hBN bilayer heterojunction quantum dots with four different radii (r1,r2,r3,r4).The dark blue, blue, green, and yello w color schemes represent charge state numbers of 0, 1, 2, and 3, respectively.

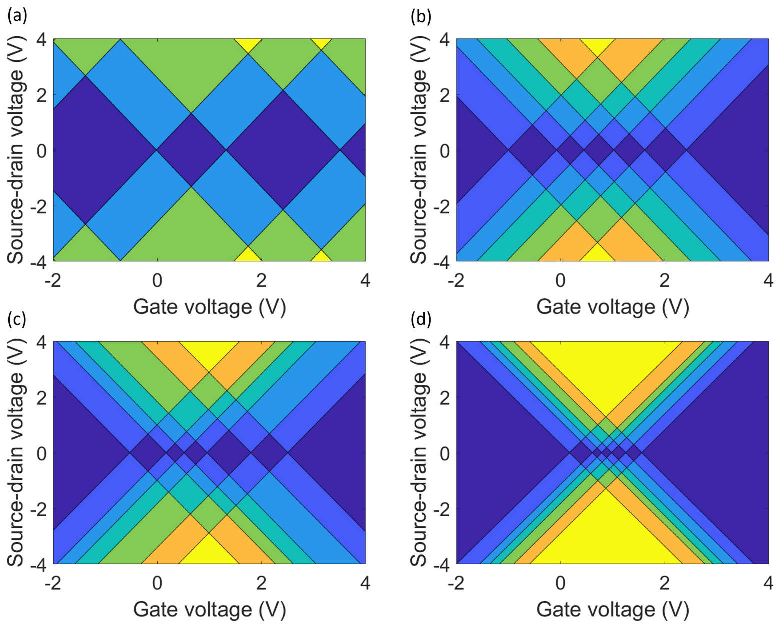

Figure S48: The charge stability plots of unrotated BA-stacked TG/hBN bilayer heterojunction quantum dots with four different radii (r1,r2,r3,r4).The dark blue, blue, green, and yellow color schemes represent charge state numbers of 0, 1, 2, and 3, respectively.

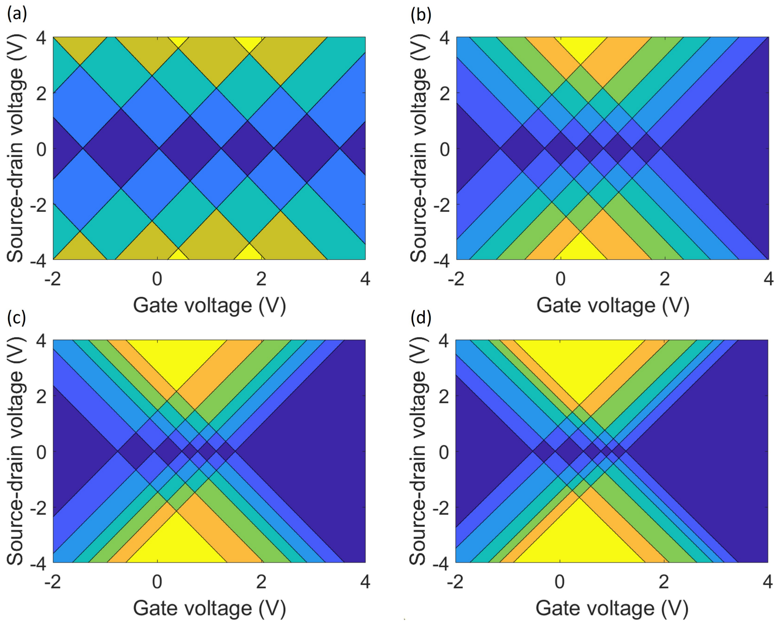

Figure S49: The charge stability plots of 28.05° rotated AA-stacked TG/hBN bilayer heterojunction quantum dots with four different radii (r1,r2,r3,r4). The dark blue, blue, green, and yellow color schemes represent charge state numbers of 0, 1, 2, and 3, respectively.

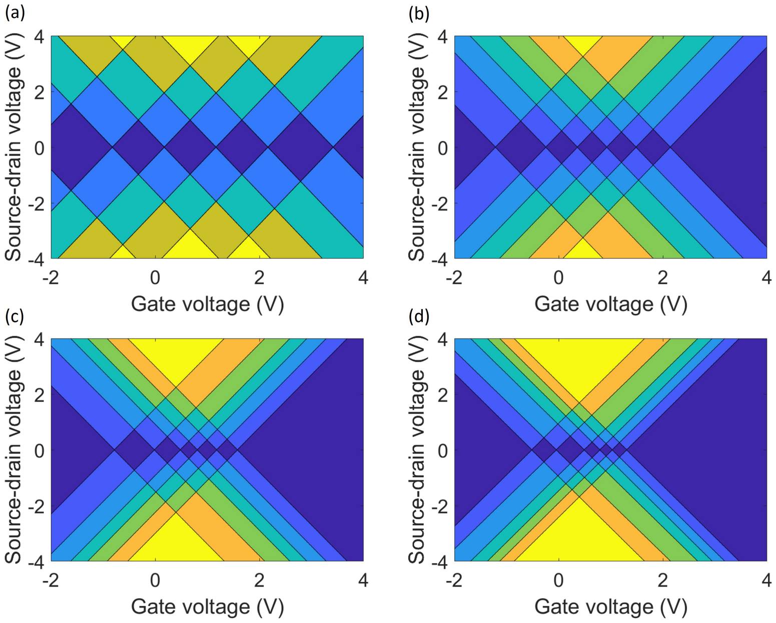

Figure S50: The charge stability plots of 28.05° rotated AB-stacked TG/hBN bilayer heterojunction quantum dots with four different radii (r1,r2,r3,r4). The dark blue, blue, green, and yellow color schemes represent charge state numbers of 0,1,2,3, respectively.

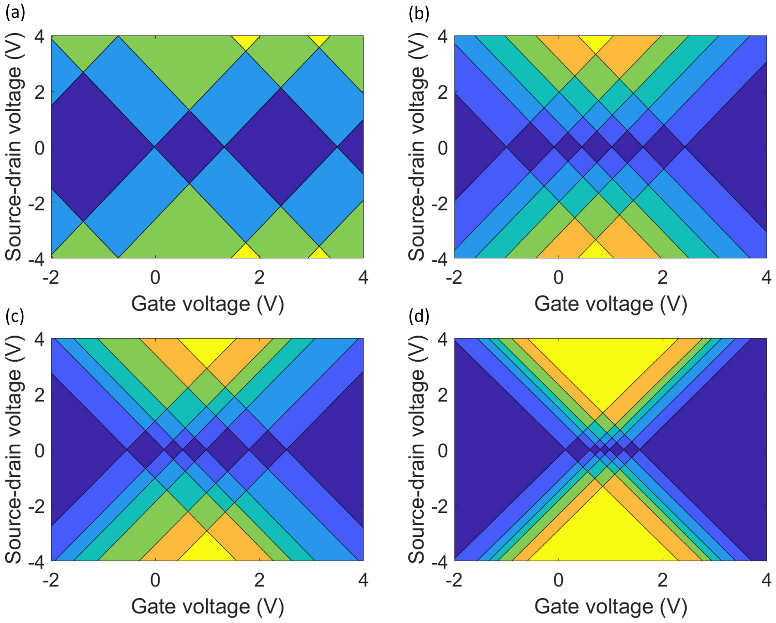

Figure S51: The charge stability plots of 28.05° rotated BA-stacked TG/hBN bilayer heterojunction quantum dots with four different radii (r1,r2,r3,r4). The dark blue, blue, green, and yellow color schemes represent charge state numbers of 0,1,2,3, respectively.

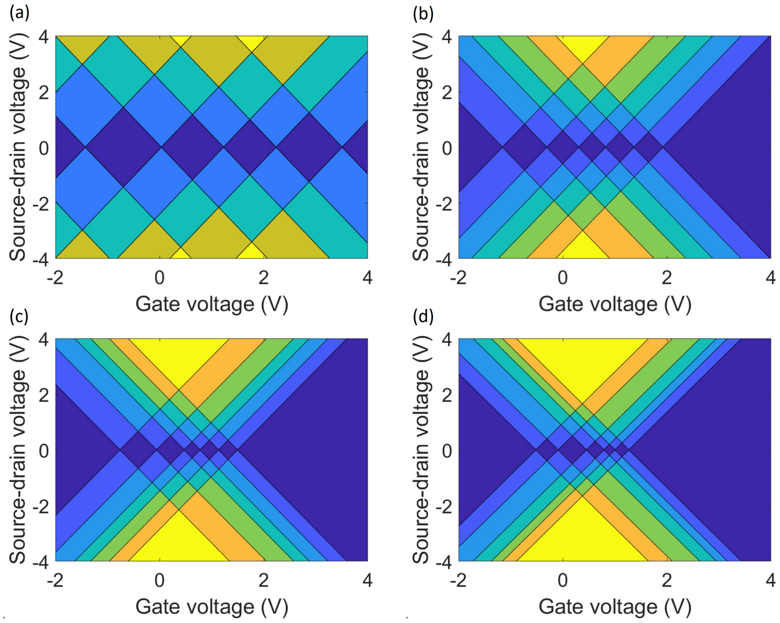

Figure S52: The charge stability plots of 25.97° rotated AA-stacked TG/hBN bilayer heterojunction quantum dots with four different radii (r1,r2,r3,r4). The dark blue, blue, green, and yellow color schemes represent charge state numbers of 0,1,2,3, respectively.

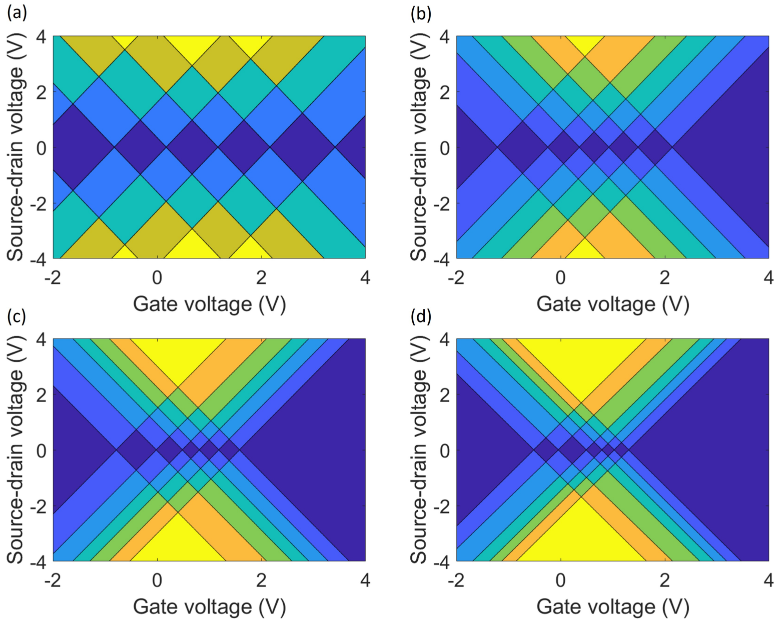

Figure S53: The charge stability plots of 25.97° rotated AB-stacked TG/hBN bilayer heterojunction quantum dots with four different radii (r1,r2,r3,r4). The dark blue, blue, green, and yellow color schemes represent charge state numbers of 0,1,2,3, respectively.

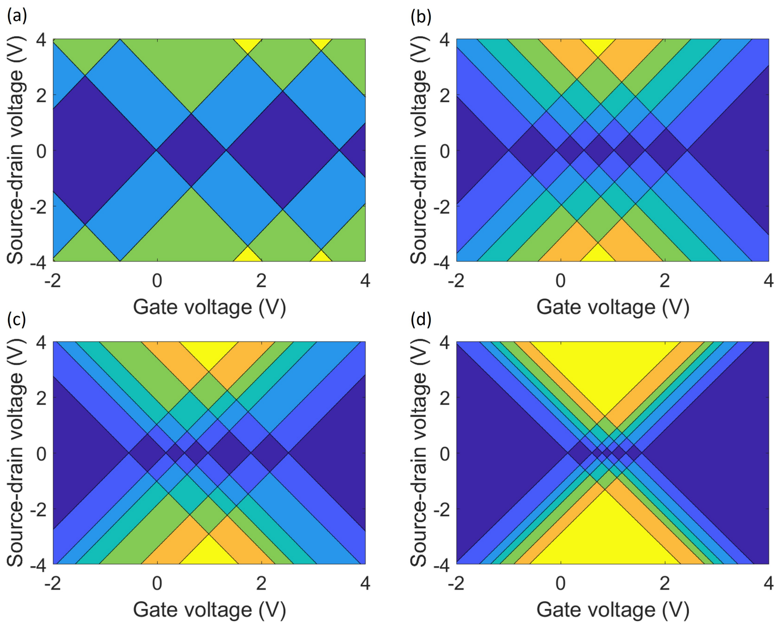

Figure S54: The charge stability plots of 25.97° rotated BA-stacked TG/hBN bilayer heterojunction quantum dots with four different radii (r1,r2,r3,r4). The dark blue, blue, green, and yellow color schemes represent charge state numbers of 0,1,2,3, respectively.

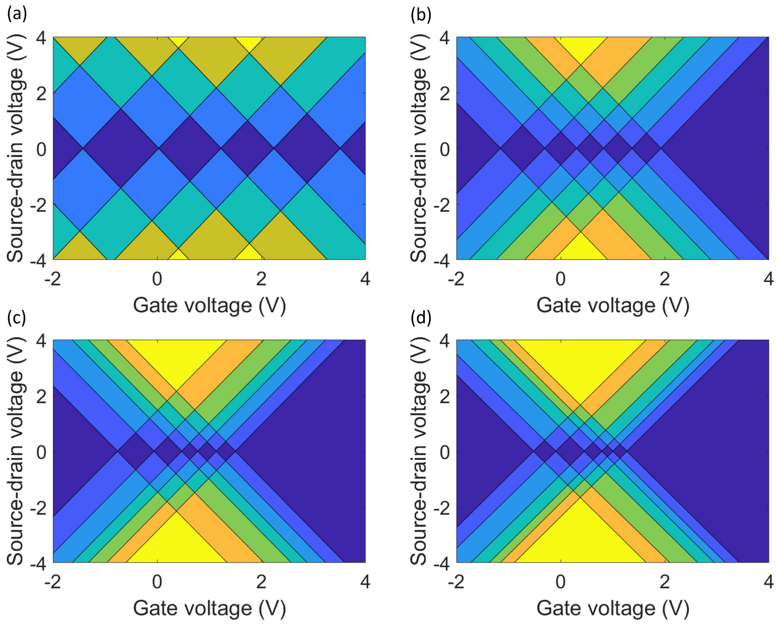

Figure S55: The charge stability plots of 21.54° rotated AA-stacked TG/hBN bilayer heterojunction quantum dots with four different radii (r1,r2,r3,r4). The dark blue, blue, green, and yellow color schemes represent charge state numbers of 0,1,2,3, respectively.

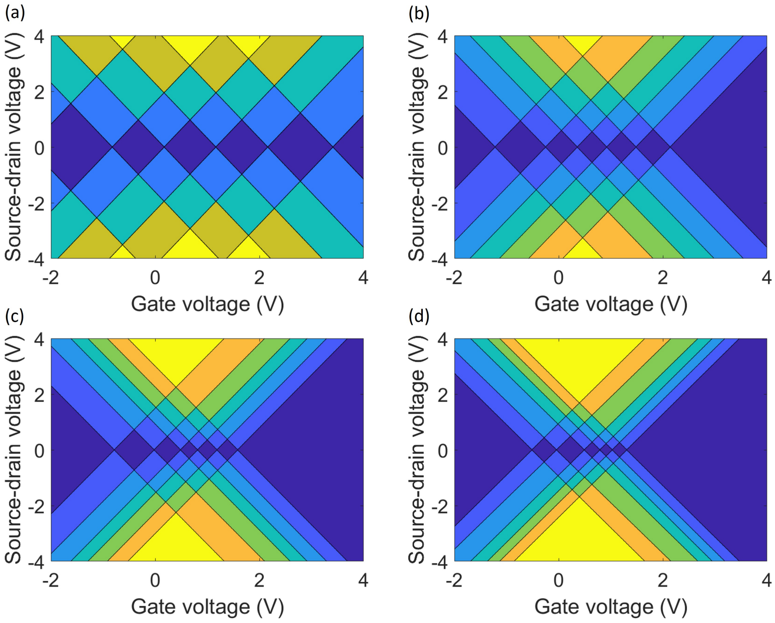

Figure S56: The charge stability plots of 21.54° rotated AB-stacked TG/hBN bilayer heterojunction quantum dots with four different radii (r1,r2,r3,r4). The dark blue, blue, green, and yellow color schemes represent charge state numbers of 0,1,2,3, respectively.

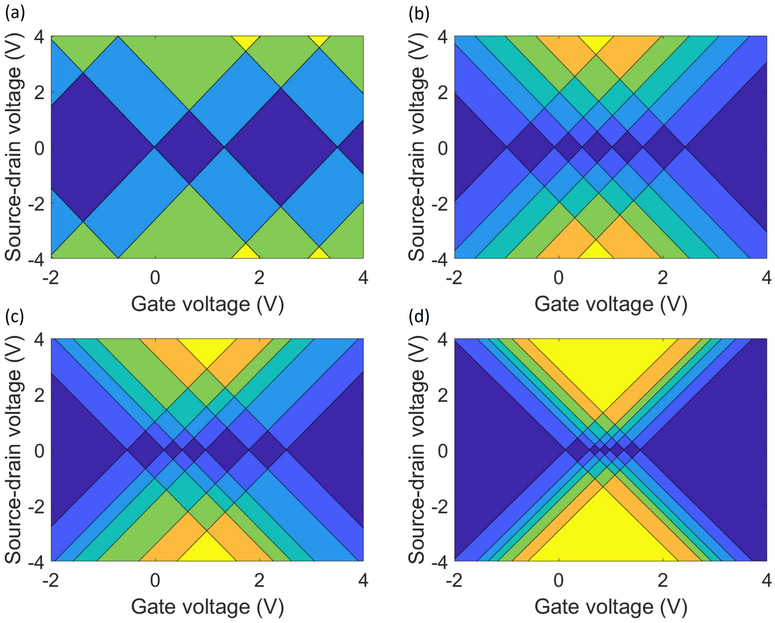

Figure S57: The charge stability plots of 21.54° rotated BA-stacked TG/hBN bilayer heterojunction quantum dots with four different radii (r1,r2,r3,r4). The dark blue, blue, green, and yellow color schemes represent charge state numbers of 0,1,2,3, respectively.

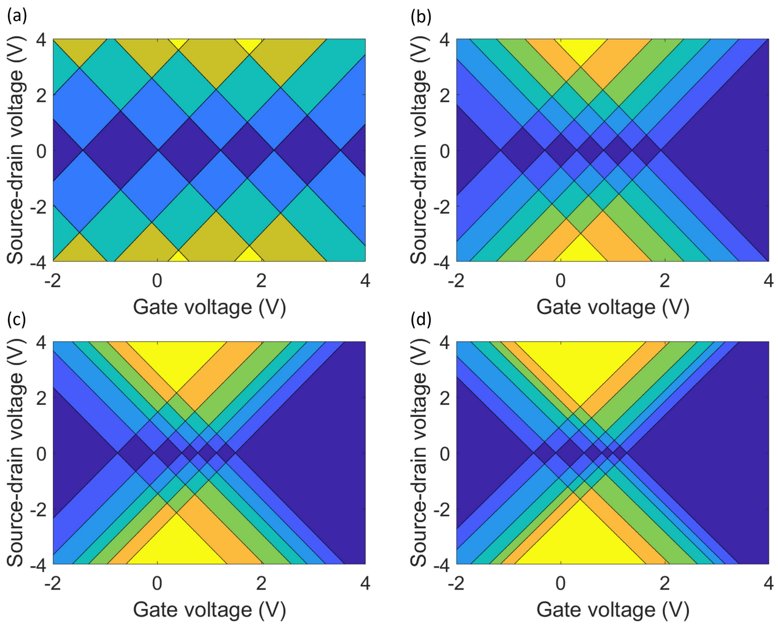

Figure S58: The charge stability plots of 17.05° rotated AA-stacked TG/hBN bilayer heterojunction quantum dots with four different radii (r1,r2,r3,r4). The dark blue, blue, green, and yellow color schemes represent charge state numbers of 0,1,2,3, respectively.

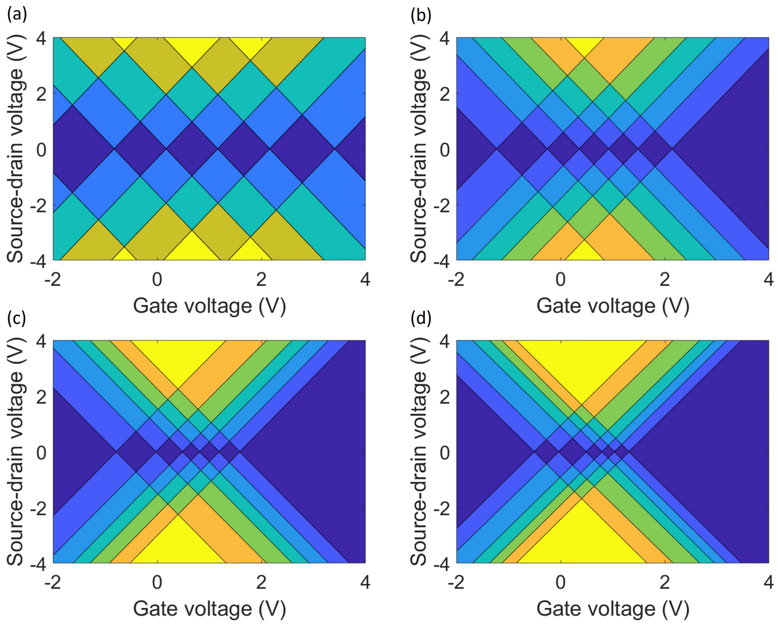

Figure S59: The charge stability plots of 17.05° rotated AB -stacked TG/hBN bilayer heterojunction quantum dots with four different radii (r1,r2,r3,r4). The dark blue, blue, green, and yellow color schemes represent charge state numbers of 0,1,2,3, respectively.

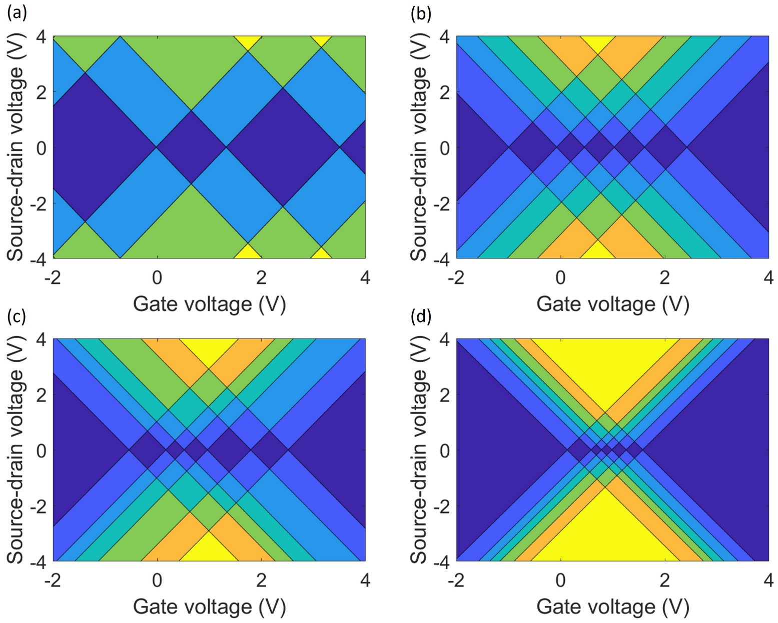

Figure S60: The charge stability plots of 17.05° rotated BA-stacked TG/hBN bilayer heterojunction quantum dots with four different radii (r1,r2,r3,r4). The dark blue, blue, green, and yellow color schemes represent charge state numbers of 0,1,2,3, respectively.

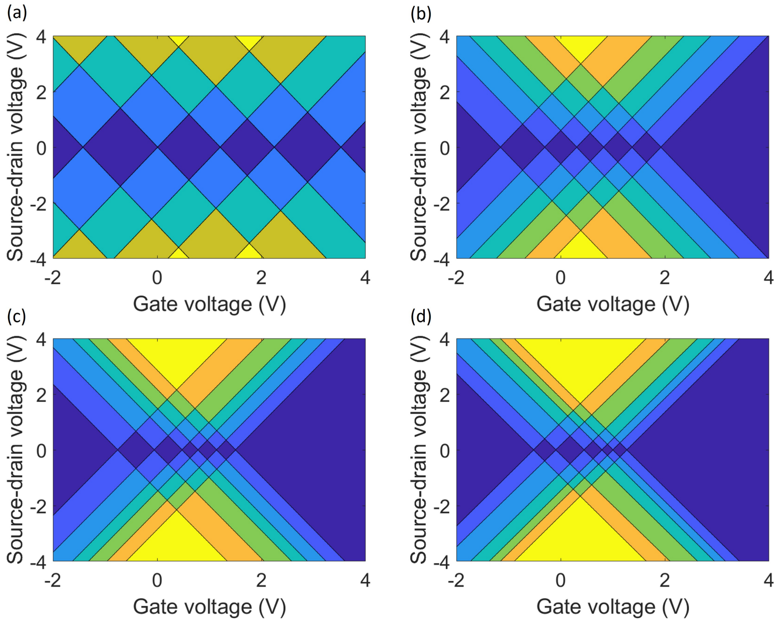

Figure S61: The charge stability plots of 11.99° rotated AA-stacked TG/hBN bilayer heterojunction quantum dots with four different radii (r1,r2,r3,r4). The dark blue, blue, green, and yellow color schemes represent charge state numbers of 0,1,2,3, respectively.

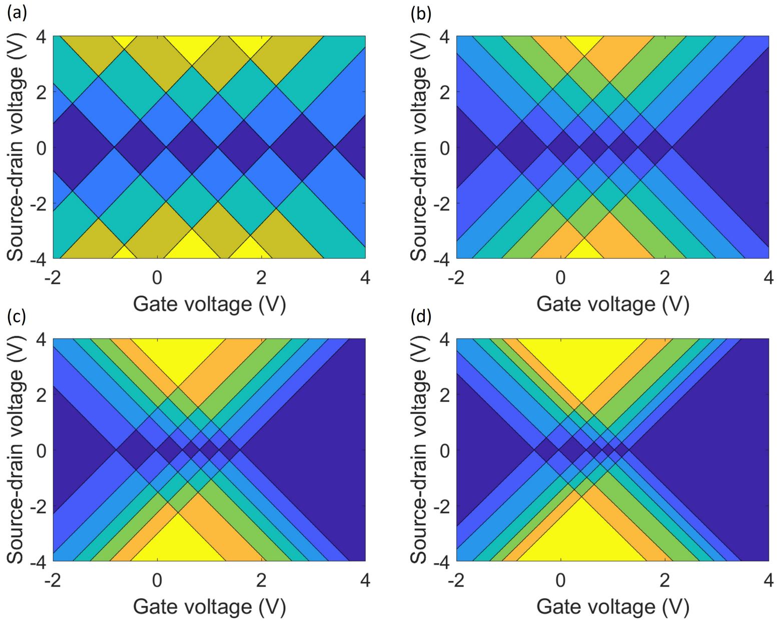

Figure S62: The charge stability plots of 11.99° rotated AB-stacked TG/hBN bilayer heterojunction quantum dots with four different radii (r1,r2,r3,r4). The dark blue, blue, green, and yellow color schemes represent charge state numbers of 0,1,2,3, respectively.

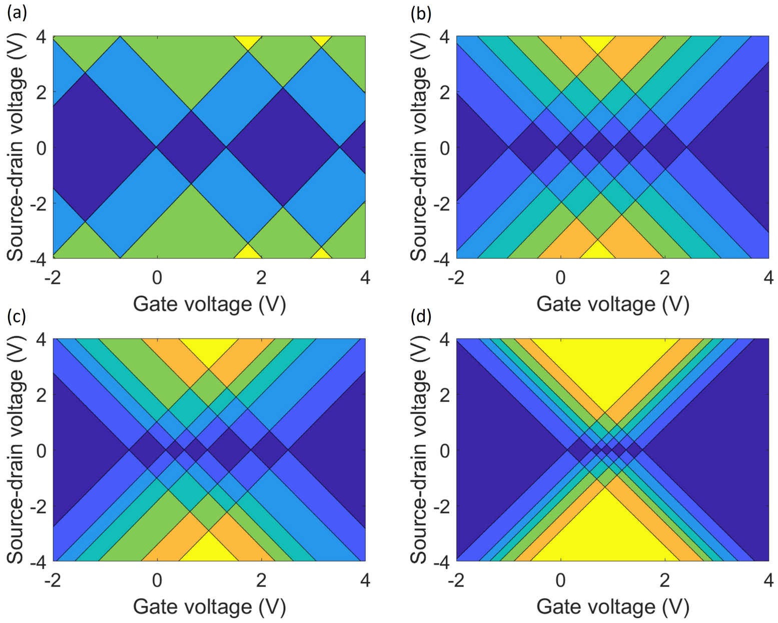

Figure S63: The charge stability plots of 11.99° rotated BA-stacked TG/hBN bilayer heterojunction quantum dots with four different radii (r1,r2,r3,r4). The dark blue, blue, green, and yellow color schemes represent charge state numbers of 0,1,2,3, respectively.

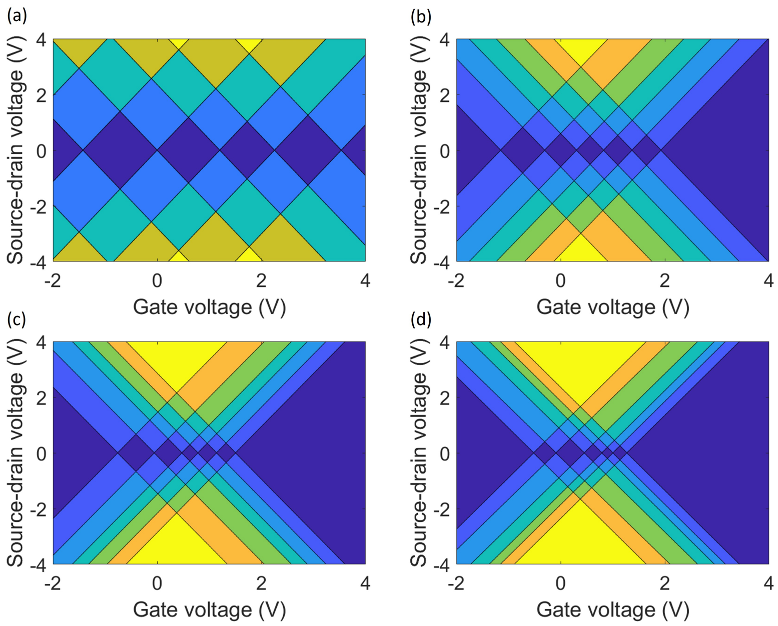

Figure S64: The charge stability plots of 6.07° rotated AA-stacked TG/hBN bilayer heterojunction quantum dots with four different radii (r1,r2,r3,r4). The dark blue, blue, green, and yellow color schemes represent charge state numbers of 0,1,2,3, respectively.

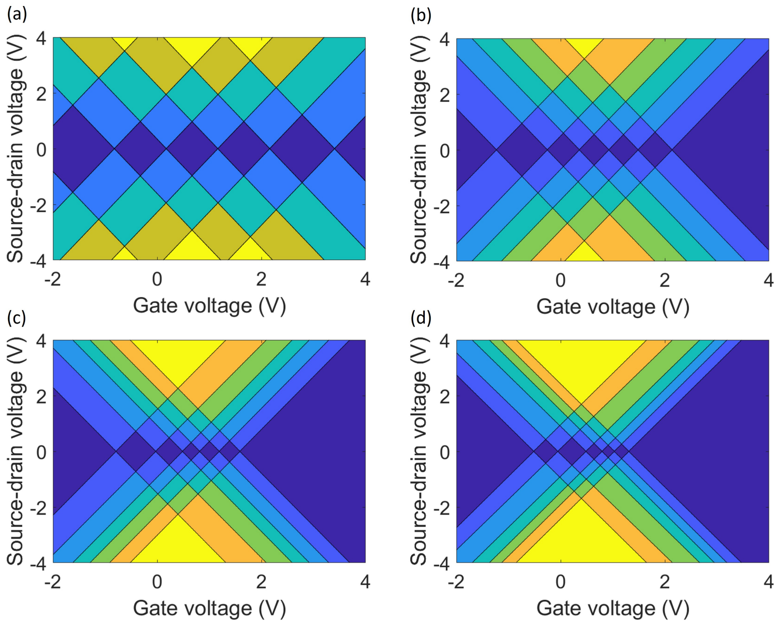

Figure S65: The charge stability plots of 6.07° rotated AB-stacked TG/hBN bilayer heterojunction quantum dots with four different radii (r1,r2,r3,r4). The dark blue, blue, green, and yellow color schemes represent charge state numbers of 0,1,2,3, respectively.

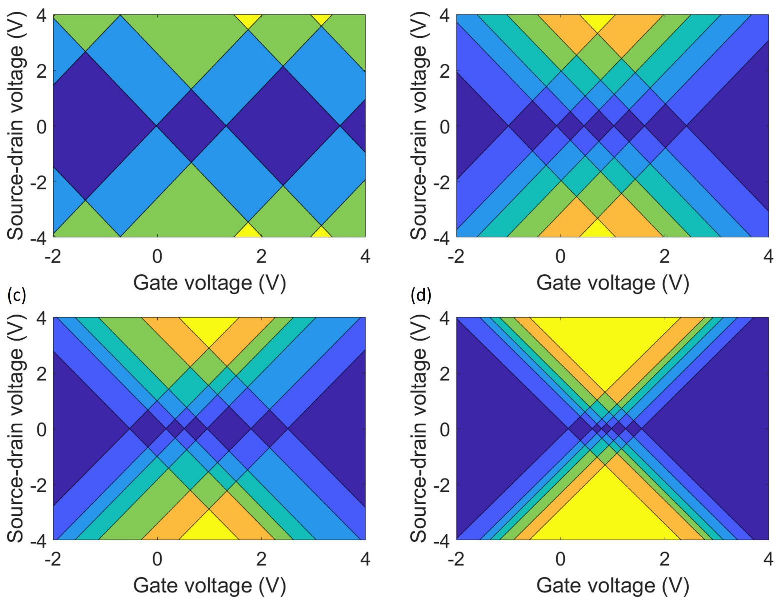

Figure S66: The charge stability plots of 6.07° rotated BA-stacked TG/hBN bilayer heterojunction quantum dots with four different radii (r1,r2,r3,r4). The dark blue, blue, green, and yellow color schemes represent charge state numbers of 0,1,2,3, respectively.

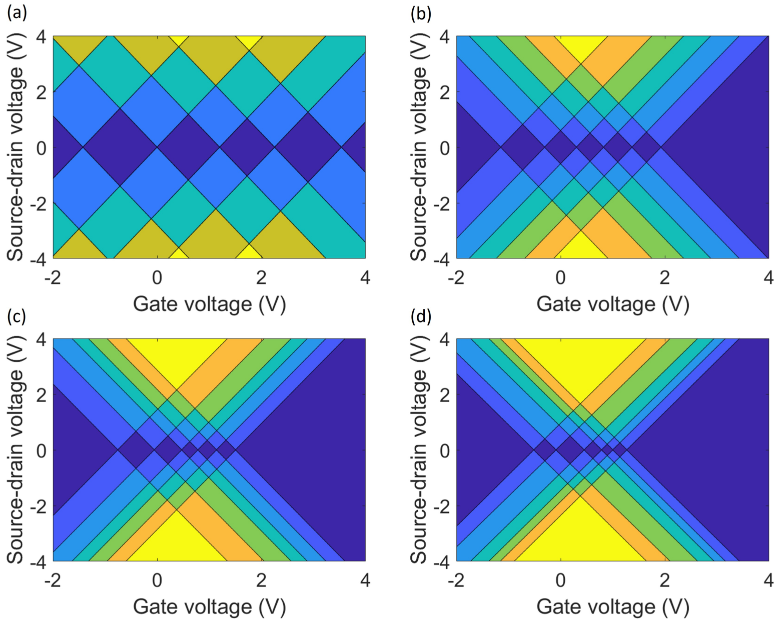

Figure S67: The charge stability plots of 2.65° rotated AA-stacked TG/hBN bilayer heterojunction quantum dots with four different radii (r1,r2,r3,r4). The dark blue, blue, green, and yellow color schemes represent charge state numbers of 0,1,2,3, respectively.

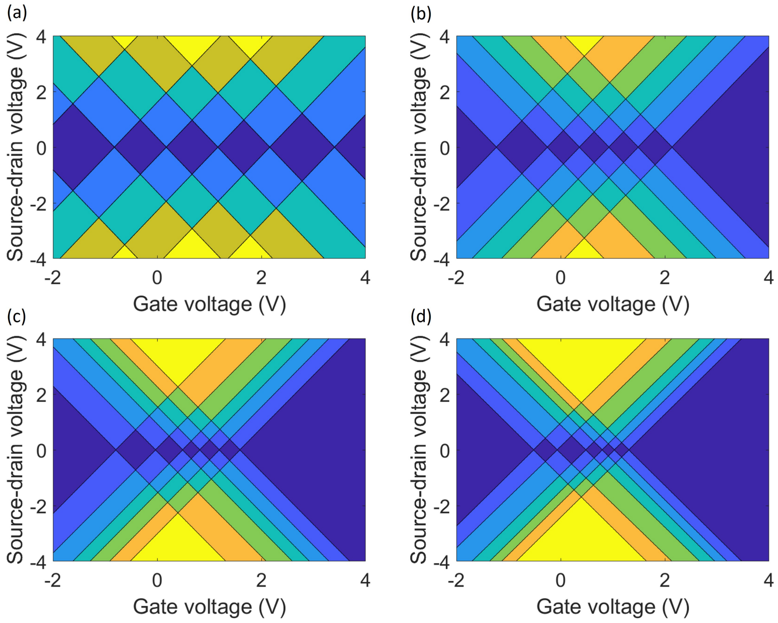

Figure S68: The charge stability plots of 2.65° rotated AB-stacked TG/hBN bilayer heterojunction quantum dots with four different radii (r1,r2,r3,r4). The dark blue, blue, green, and yellow color schemes represent charge state numbers of 0,1,2,3, respectively.

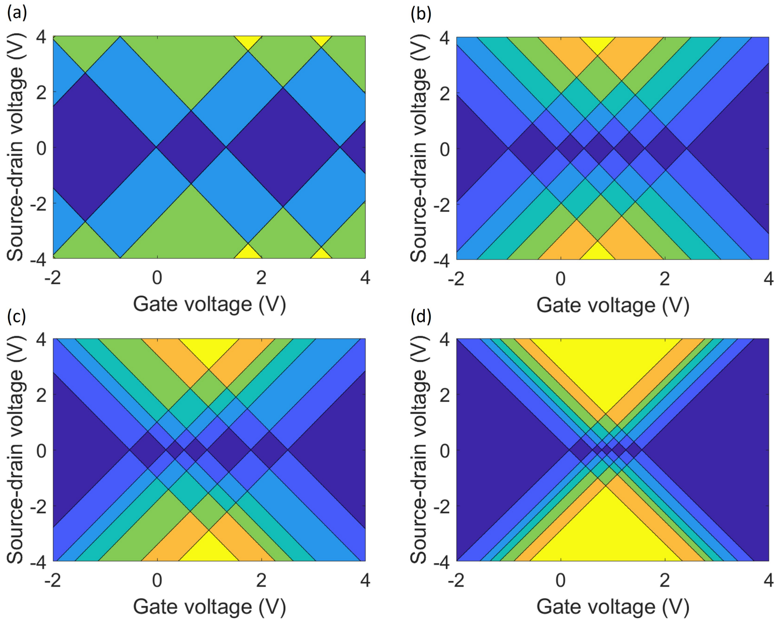

Figure S69: The charge stability plots of 2.65° rotated BA-stacked TG/hBN bilayer heterojunction quantum dots with four different radii (r1,r2,r3,r4). The dark blue, blue, green, and yellow color schemes represent charge state numbers of 0,1,2,3, respectively.

Section VI.The gate coupling strengths of TG/hBN island quantum dots in AB and BA stacking configurations for three radii (r_2, r_3, r_4)

Table S1: The linear gate coupling strengths between the gates and TG/hBN island quantum dots in double-gated single-electron transistor configurations with three different radii (r_2, r_3, r_4) for AB stacking

rotation	Linear gate-island coupling strength(α)		
angle	r_2	r_3	r_4
0°	0.967558	0.946150	0.917978
28.05°	0.967980	0.947133	0.919474
25.97°	0.967960	0.947076	0.919474
21.54°	0.967909	0.946953	0.919287
17.05°	0.967848	0.946775	0.919057
11.99°	0.967776	0.946602	0.918764
6.07°	0.967685	0.946391	0.918375
2.65°	0.967613	0.946268	0.918146

Table S2: The quadratic gate coupling strengths between the gates and TG/hBN island quantum dots in double-gated single-electron transistor configurations with three different radii (r_2, r_3, r_4) for AB stacking

rotation	Linear gate-island coupling strength($\beta(eV^{-1})$		
angle	r_1	r_2	r_3
0°	-0.000312	-0.002544	-0.011802
28.05°	-0.000281	-0.002346	-0.010662
25.97°	-0.000282	-0.002358	-0.010674
21.54°	-0.000286	-0.002384	-0.010833
17.05°	-0.000291	-0.002411	-0.011025
11.99°	-0.000297	-0.002444	-0.011267
6.07°	-0.000303	-0.002489	-0.011546
2.65°	-0.000308	-0.002518	-0.011694

Table S3: The linear gate coupling strengths between the gates and TG/hBN island quantum dots in double-gated single-electron transistor configurations with three different radii (r_2, r_3, r_4) for BA stacking

rotation	Linear gate-island coupling strength(α)		
angle	r_2	r_3	r_4
0°	0.969426	0.949691	0.926176
28.05°	0.969440	0.949401	0.925669
25.97°	0.969450	0.949443	0.925863
21.54°	0.969458	0.949518	0.926242
17.05°	0.969455	0.949575	0.926188
11.99°	0.969431	0.949638	0.926301
6.07°	0.969417	0.949700	0.926344
2.65°	0.969420	0.949710	0.926242

Even if one considers a non-zero source/drain capacitance (Cs, Cd), in the sequential tunneling regime its main effect would be to introduce a small, constant offset in the gate-voltage axis of the stability diagram, without altering the relative positions of thresholds or the comparative trends (e.g., the hierarchy of size > stacking > twist angle) reported in this work. A simple estimate using a parallel-plate capacitor model suggests that for realistic geometric factors (Cs,d/Cg <1), this offset is significantly smaller than the gate-voltage shifts induced by the variation in quantum dot size or stacking configuration, which are the primary focus of our conclusions.

Section VI.The gate coupling strengths of TG/hBN island quantum dots in AB and BA stacking configurations for three radii (r_2, r_3, r_4)

Table S4: The quadratic gate coupling strengths between the gates and TG/hBN island quantum dots in double-gated single-electron transistor configurations with three different radii (r_2, r_3, r_4) for BA stacking

rotation angle	Linear gate-island coupling strength($\beta(eV^{-1})$		
rotation angle	Quadratic gate-island coupling strength(β (eV^{-1}))		
	r_1	r_2	r_3
0°	-0.000252	-0.002096	-0.010033
28.05°	-0.000256	-0.002143	-0.010184
25.97°	-0.000255	-0.002137	-0.010046
21.54°	-0.000254	-0.002124	-0.010018
17.05°	-0.000253	-0.002111	-0.009907
11.99°	-0.000252	-0.002099	-0.010897
6.07°	-0.000252	-0.002092	-0.010007
2.65°	-0.000252	-0.002091	-0.010018
